# Immunomodulation of inflammatory responses preserves retinal integrity in murine models of pericyte-depletion retinopathy

**DOI:** 10.1172/jci.insight.184465

**Published:** 2025-07-01

**Authors:** Urbanus Muthai Kinuthia, Christoph Moehle, Ralf H. Adams, Thomas Langmann

**Affiliations:** 1 Laboratory for Experimental Immunology of the Eye, Department of Ophthalmology, University of Cologne, Faculty of Medicine and University Hospital Cologne, Cologne, Germany.; 2 Centre for Molecular Medicine Cologne (CMMC), University of Cologne, Cologne, Germany.; 3 Center of Excellence for Fluorescent Bioanalytics, University of Regensburg, Regensburg, Germany.; 4 Department of Tissue Morphogenesis, Max Planck Institute for Molecular Biomedicine and University of Münster, Faculty of Medicine, Münster, Germany.

**Keywords:** Immunology, Ophthalmology, Cellular immune response, Diabetes, Retinopathy

## Abstract

The loss of integrity of the blood retina barrier (BRB) is a key pathological hallmark of vision-threatening complications in diabetic retinopathy (DR). Although DR is considered a microvascular disease, mounting evidence from mouse models and patients show that inflammation is closely connected with microvasculopathy. Inflammatory responses during retinal pathophysiology are often orchestrated by microglia, resident innate immune cells of the retina. However, the precise role of microglia activity during DR pathogenesis remains elusive. Here, we used an anti-PDGFRβ antibody and inducible endothelial cell–specific PDGFB-KO during postnatal development of retinal vasculature to reproduce a key feature of DR pathology in mice. In addition, we applied a minocycline therapy to modulate retinal inflammation. Postnatal depletion of pericytes or loss of PDGFB in retinal vessels altered BRB integrity and triggered secretion of angiogenic and inflammatory factors with concomitant microglia reactivity, which was sustained in retinas of adult mice. Microglia reactivity was accompanied by upregulation of disease-associated genes. Notably, minocycline attenuated the cycle of inflammatory responses in young and mature retinas, thereby preserving retinal vascular and structural integrity in mice. Together, our findings suggest that immunomodulation of microglia-driven inflammatory responses preserves retinal vasculature and maintains BRB integrity in 2 different mouse models of human DR.

## Abstract 2

This study reports on key inflammatory processes and a microglia-targeting therapy approach in mouse models of diabetic retinopathy, a vision-threatening complication of diabetes mellitus.

## Graphical abstract

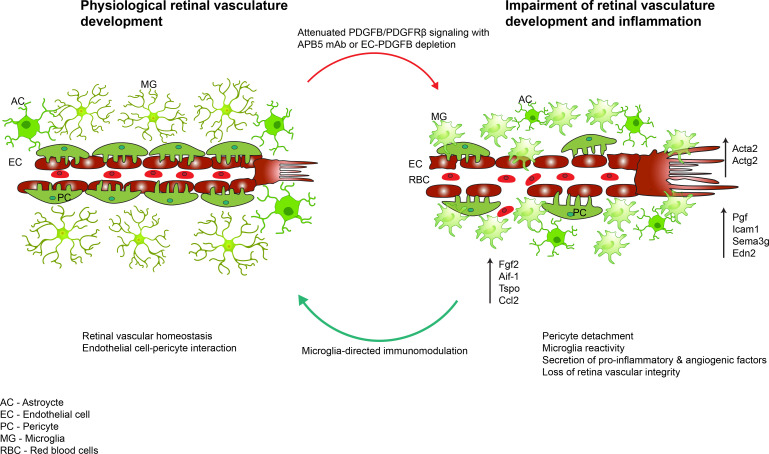

## Introduction

In the developing central nervous system (CNS), pericytes and endothelial cells form a physiological barrier that not only protects the CNS parenchyma but also acts as a molecular sieve that regulates passage of fluids and ions between systemic circulation and CNS tissues (1). The anatomical barriers namely, blood brain barrier (BBB) and blood retina barrier (BRB), help maintain tissue physiological properties and protect neurons and glial cells from foreign DAMPs (toxins) (2, 3). The BRB is divided into 2 parts, an inner barrier that is composed of a network of pericytes, endothelial cells (ECs), and Müller glial cells and an outer retina consisting of retinal pigment epithelial (RPE) cells (3). The development and formation of a functional BRB occurs through vasculogenesis and angiogenic sprouting during embryonic and postnatal life in humans and mice, respectively (4, 5). The angiogenic penetration of the developing retinal parenchyma is regulated by neural-vascular interactions involving signaling among Müller cells, astrocytes, neurons (ganglion cells, amacrine cells and horizontal cell), microglia, and ECs (5, 6).

A compromised BRB is associated with metabolic disorders such as diabetes mellitus (DM) and leads to immune activation and chronic inflammation, which drive retinal pathologies including diabetic retinopathy (DR). Clinically, DR is divided into 2 broad forms; an early nonproliferative form (NPDR) characterized by pericyte loss, vascular permeability, capillary occlusion, and basement membrane thickening and an advanced proliferative form (PDR) featuring vitreous hemorrhage, pathological neovascularization, and retinal detachment. A key pathological feature common to both forms of DR is diabetic macula edema (DME), which is a consequence of BRB breakdown leading to intraretinal plasma leakage, retinal swelling, and vision loss (7).

Currently, anti–vascular endothelial growth factor (VEGF) therapies that regress neovascularization and DME are the gold standard of care for patients with DR. The anti-VEGF therapy with aflibercept has previously demonstrated clinical improvement of vision among patients with DME (8). A major challenge with anti-VEGF therapy is that approximately 40%–50% of patients with DR fail to show clinical improvement following anti-VEGF therapy (9, 10), thus necessitating the search for therapeutic agents for the nonresponding patients. Recently, therapeutic targeting of senescent vasculature in patients with advanced DME with a senolytic small molecule inhibitor, BCL-xL, provided promising findings in a phase 1 clinical trial (11), but additional large clinical trials are needed to verify the beneficial effects of senolytic agents in DR.

While DR is a consequence of sustained hyperglycemia due to DM, an underlying feature of DR pathogenesis is microglia activation. Microglia cells are the immune sentinels of the CNS and retina. Under homeostatic conditions, microglia possess a ramified morphology and protect the retinal microenvironment from endogenous and exogenous noxious stimuli. In patients with DR, microglia activation occurs at different stages of the disease. In NPDR, there is a moderate increase in the number of reactive microglia around dilated vessels and microaneurysms, whereas in PDR, hypertrophic microglia cluster around cotton-wool spots and areas of retinal neovascularization and infiltrate the outer retina, subretinal space (SRS), and optic nerve in DME thereby propagating inflammatory responses (12). In addition, there exists a positive correlation between serum markers of microglia activation in hyperreflective retinal spots in patients with NPDR and PDR (13). Similarly, microglia activation, hypertrophy, and dysregulation of the CD200/CD200R signaling immune checkpoint in microglia is a prominent feature of experimental DR (14–17).

Microglia orchestrate a chronic low-grade inflammation that severely affects retinal neurons, aggravating EC apoptosis and thinning the outer nuclear layer of the retina. However, owing to the lack of models that recapitulate the ocular phenotype seen in patients with DR, the role of microglia-mediated inflammatory responses in advanced DR remains unresolved.

In this study, we sought to investigate the cellular and molecular characteristics of retinal inflammation, with a focus on microglia and the vasculature, in 2 mouse models that reproduce key features of the human disease. The first experimental model was generated by inhibition of PDGFB/PDGFRβ signaling in the postnatal retina using an anti-PDGFRβ mAb (18) and reproduces important pathophysiological features of DR, including pathological angiogenesis, leaky vasculature, microglia activation, and fast photoreceptor demise in adult mice. The second mouse model, *Pdgfb*^iECKO^, was generated via tamoxifen-inducible depletion of PDGFB in retinal endothelium under control of VE-Cadherin promoter (19) and recapitulates important aspects of the human disease, including retinal vascular aneurysms and leakage as well as microglia activation, and gradual loss of photoreceptors. Both models were treated with minocycline, a second-generation tetracycline that penetrates the blood barriers and exerts beneficial pleiotropic effects besides its antibacteriostatic effect. (20). Previous studies, including our own, have demonstrated minocycline’s potential to limit overt microglia activation while preserving the CNS neurons and photoreceptors in models of retinal pathologies (21–25). Herein, we investigated the therapeutic benefit of minocycline in the modulation of inflammatory responses both at the onset and in progression of disease in mouse models of pericyte-depletion retinopathy.

## Results

### Inhibition of pericyte recruitment alters the structural composition of inner blood retina barrier in P10 mice.

In mice, unlike in humans, the development of the retinal vasculature occurs in postnatal life beginning at P1 with the formation of the superficial vascular plexus, which starts to sprout vertically at P7–P8 to form the deeper plexus by P10 and intermediate plexus by P12–P15 (26). PDGFRβ expressing pericytes are recruited to PDGFB expressing ECs via PDGFB/PDGFRB signaling, which is indispensable for maintenance of vascular integrity and function in postnatal murine retina (18, 27). Here, we investigated the cellular changes of the inner BRB and resident immune cells upon impairment of pericyte recruitment to growing ECs, and we assessed the therapeutic effect of minocycline the vasculature and microglia in postnatal life.

**Figure 1:**
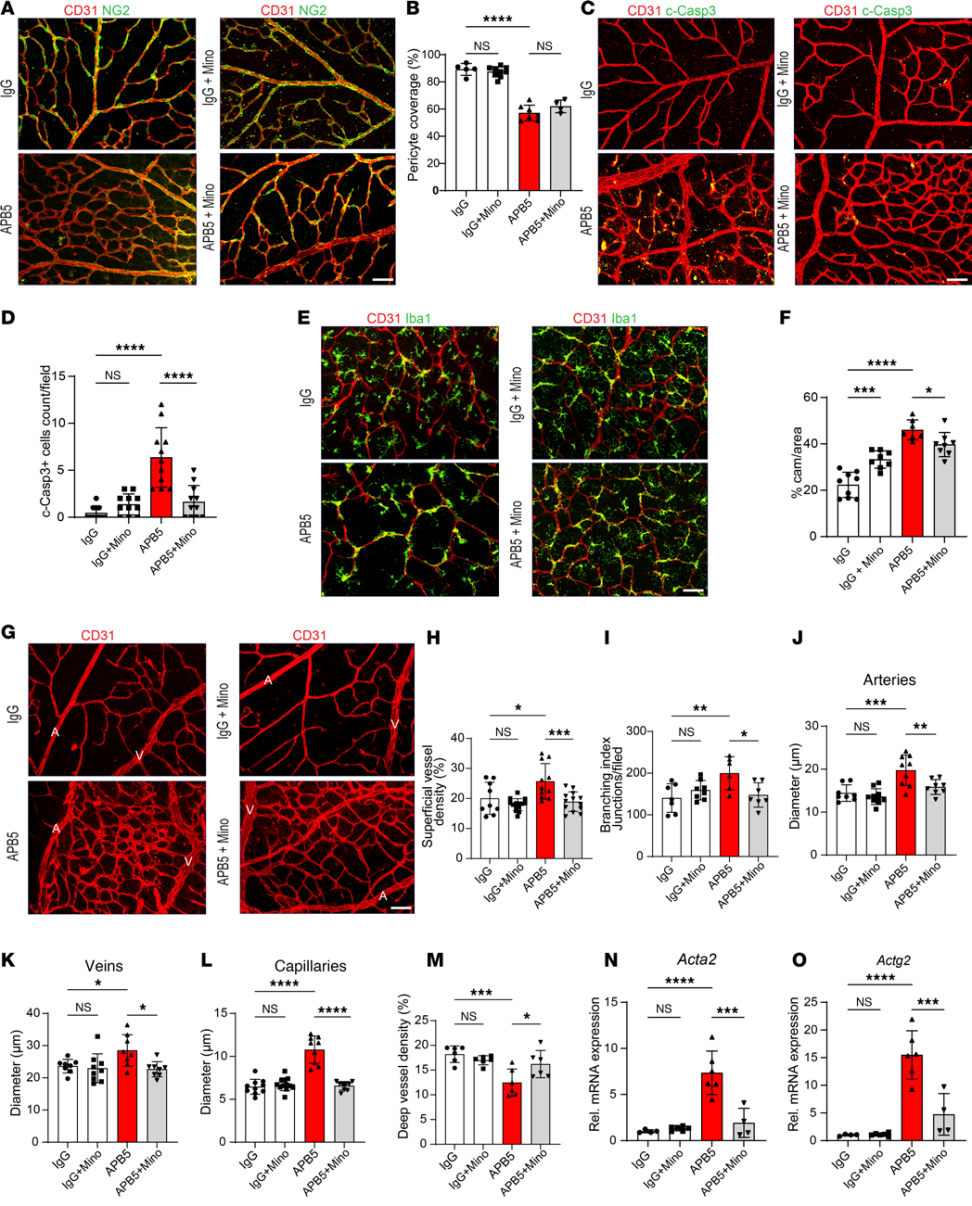
Changes in the structural components of the inner blood retina barrier during postnatal retinal vascular development at P10 following treatment with IgG or APB5 at P1 via s.c injection and daily administration of minocycline via i.p (P5–P9). (**A** and **B**) IHC for CD31 and NG2 in the capillary plexus and quantification of pericyte coverage (*n* = 4–6 retinas). (**C** and **D**) IHC and quantification of CD31 and cleaved caspase-3^+^ cells in the superficial plexus (*n* = 11). (**E** and **F**) IHC of CD31 and Iba1 in the deep vascular plexus and quantification of capillary-associated microglia (CAM) (*n* = 7-9 retinas). (**G** and **H**) Representative images IHC for CD31 on the superficial vascular plexus with the arteries and veins — labeled as A and V, respectively — and quantification of superficial vascular density (*n* = 9–12 retinas). (**I**–**L**) Quantification of the branching index of superficial vasculature (**I**) and diameters for arteries (**J**), veins (**K**), capillaries (**L**), and deep vascular density (**M**) (*n* = 6–8 retinas). (**N** and **O**) Quantification of relative mRNA expression levels of smooth muscle genes *Acta2* (**N**) and *Actg2* (**O**) in the retina (*n* = 4–6 retinas). Scale bar: 50 μm. *P < 0.05, **P < 0.01, ***P < 0.001, ****P < 0.0001. Data are shown as mean ± SD. One-way ANOVA.

In retinas of WT mice treated with IgG, pericytes were adequately present on the ECs in the capillary plexus at P10. In contrast, pericyte coverage of the superficial capillary plexus was impaired in the retinas of mouse pups treated with 30 μg of the rat anti–mouse PDGFRβ mAb (clone APB5) via s.c. administration at P1, and this change did not differ appreciably upon daily treatment with minocycline (P5–P9) via i.p. injection (Figure 1, A and B). Since inadequate pericyte coverage exposes ECs to inflammatory stress, we tested whether apoptosis of ECs was an early feature of the APB5-induced retinopathy. Our IHC results revealed an increase in expression of cleaved caspase-3 by ECs in APB5 retinas, which were reduced in minocycline-treated mice at P10 (Figure 1, C and D, and Supplemental Figure 1, A–C; supplemental material available online with this article; https://doi.org/10.1172/jci.insight.184465DS1). Furthermore, we sought to investigate the association of microglia cells and capillaries in the deep vascular plexus. IHC analysis of ECs and Iba1^+^ cells in the deep vascular plexus showed that a significant proportion of Iba1^+^ cell bodies aligned with the vasculature in the deeper plexus of APB5-treated mice. These microglia cells, termed as capillary-associated microglia (CAM), were significantly lower in the IgG- and minocycline-treated mice (Figure 1, E and F). Consistent with the changes in pericyte coverage and inflammation, we investigated the vascular phenotype of main vessels in the P10 retinas. Besides destabilization of the superficial vasculature network leading to increased vascular density and branching index (Figure 1, G–I), APB5 induced dilation of the arteries, veins, and capillaries in the central retina at P10, which were rescued in response to minocycline treatment (Figure 1, J–L). In addition, treatment with minocycline restored the vascular density of the deep plexus to levels similar to those of control mice (Figure 1M). Since pericyte-free ECs may acquire inflammatory properties, we examined the expression levels of genes encoding smooth muscle cell contractile factors, *Acta2* and *Actg2*. Whereas the mRNA levels of *Acta2* and *Actg2* were significantly upregulated in APB5 retinas compared with control retinas, treatment with minocycline significantly reduced these levels in P10 retinas (Figure 1, N and O). Since loss of BRB integrity can trigger recruitment of circulating monocytes, we used the F4/80 macrophage marker to detect the presence of macrophages in the P10 retinas. Our analyses revealed that F4/80^+^ macrophages were present in the retinas of all mice but with a higher presence in retinas of APB-treated mice (Supplemental Figure 1, D–G). Surprisingly, the F4/80^+^ macrophages aligned the vasculature of APB5 retinas, a feature that was abrogated upon treatment with minocycline (Supplemental Figure 1H). Taken together, these findings suggest that minocycline regulated vascular remodeling and modulated macrophage infiltration in the developing murine retina upon depletion of pericytes to restore vascular integrity and function.

### Microglia reactivity is an early feature of pericyte-depletion retinopathy.

**Figure 2:**
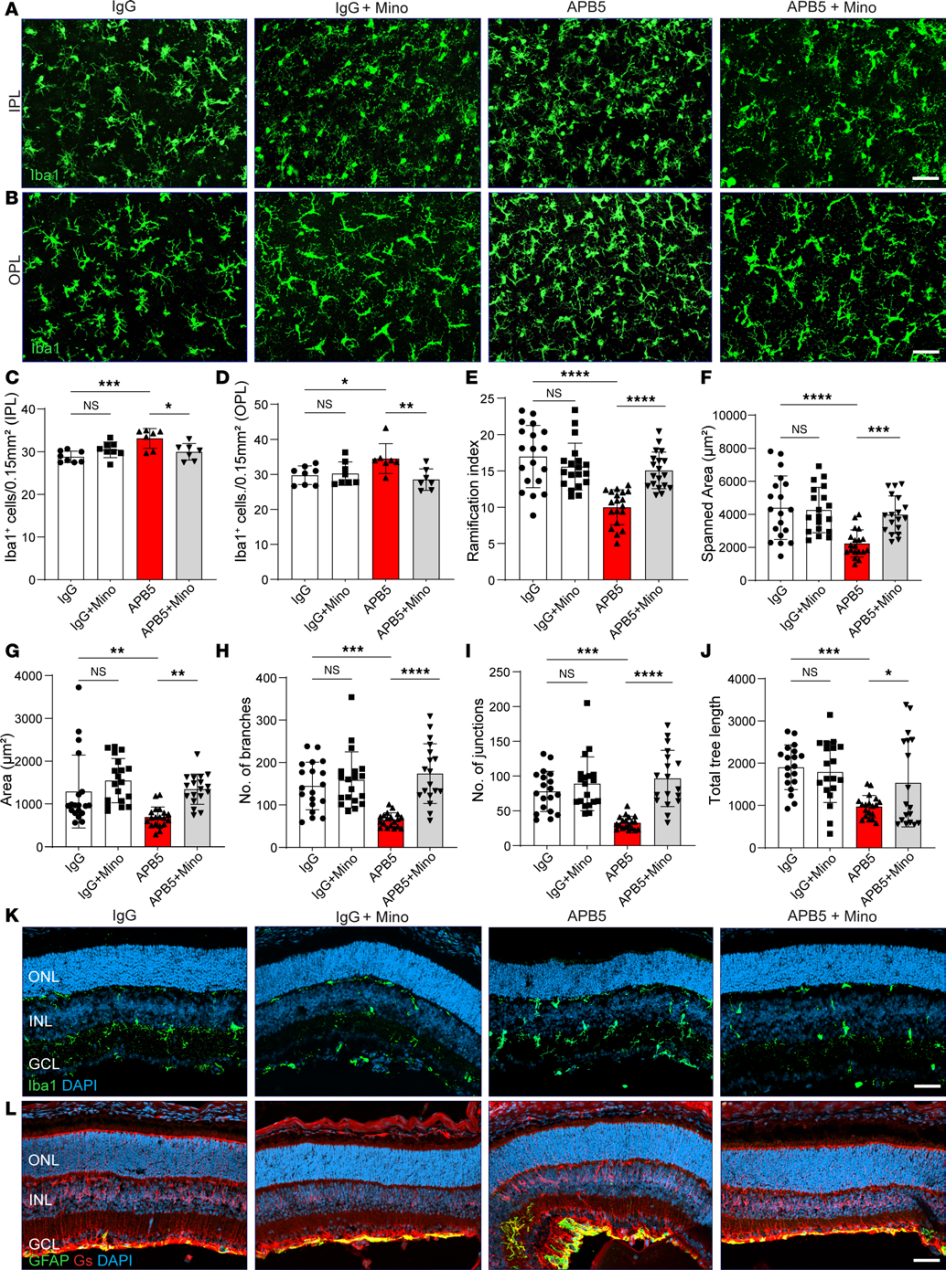
Microglia reactivity is an early event of APB5-induced retinopathy. (**A** and **B**) Representative images of Iba1^+^ cells in the IPL (**A**) and OPL (**B**) from retinal whole mounts. (**C** and **D**) Quantification of the number of Iba1^+^ cells in the IPL (**C**) and OPL (**D**) (*n* = 7–8 retinas). (**E**–**J**) Morphological analyses of retinal microglia showed that APB5 affected all analyzed parameters; ramification index (**E**), spanned area (**F**), area (**G**), number of branches (**H**), number of junctions (**I**), and total tree length (**J**) (*n* = 19 Iba1^+^ cells per group). Treatment of APB5 mice with minocycline restored the homeostatic state of microglia as indicated by all analyzed morphometric attributes. (**K**) IHC for Iba1 and DAPI on retinal sections showing the migration of Iba1^+^ cells across the INL in the APB5 retinas. (**L**) IHC for GS and GFAP on retinal sections highlighting the Müller glia reactivity in the APB5-treated retinas. Scale bar: 50 μm. *P < 0.05, ** *P* < 0.01, *** *P* < 0.001, ****P < 0.0001. Data represent mean ± SD. One way ANOVA. IPL; inner plexiform layer, OPL; outer plexiform layer; ONL, outer nuclear layer; INL, inner nuclear layer; GCL, ganglion cell layer; GS, glutamine synthetase; GFAP, glial fibrillary acid protein.

To gain insights into the cellular changes of microglia in the early stage of APB5-induced retinopathy, we used immunocytochemistry with Iba1 to stain microglia cells in P10 retinas. In contrast to control retinas, Iba1^+^ cells clustered in the inner plexiform layer (IPL) and outer plexiform layer (OPL) of APB5 retinas (Figure 2, A and B). Quantification of the number of Iba1^+^ cells in the IPL and OPL showed that minocycline decreased the number of activated microglia in the APB5-treated mice (Figure 2, C and D). Since microglia activation involves a morphological transformation from a ramified to an amoeboid form, we analyzed the morphometric attributes of microglia in the OPL. In contrast to the morphometric attributes of microglia in control retinas, microglia in retinas of APB5-treated mice showed a significant reduction in the ramification index, total area, spanned area, number of branches, number of junctions, and tree length, which improved to the homeostatic state upon treatment with minocycline (Figure 2, E–J). These findings suggest that, within the BRB, minocycline preserved the morphological features of homeostatic microglia at an early stage of retinal inflammation. Additional immunocytochemistry with Iba1 on retinal sections from P10 mice corroborated our findings of increased Iba1^+^ cells in the retinal layers of APB5-treated mice and the immunosuppressive role of minocycline (Figure 2K). Because Müller cell gliosis is a common feature of retinal pathologies, we also investigated the expression of GFAP by Müller glia in P10 retinas. The results from staining of retinal sections showed that GFAP stress fibers were developing in the APB5 retinas but not in the control or minocycline-treated groups (Figure 2L).

### Minocycline limits inflammatory and angiogenic responses in the postnatal retina.

**Figure 3:**
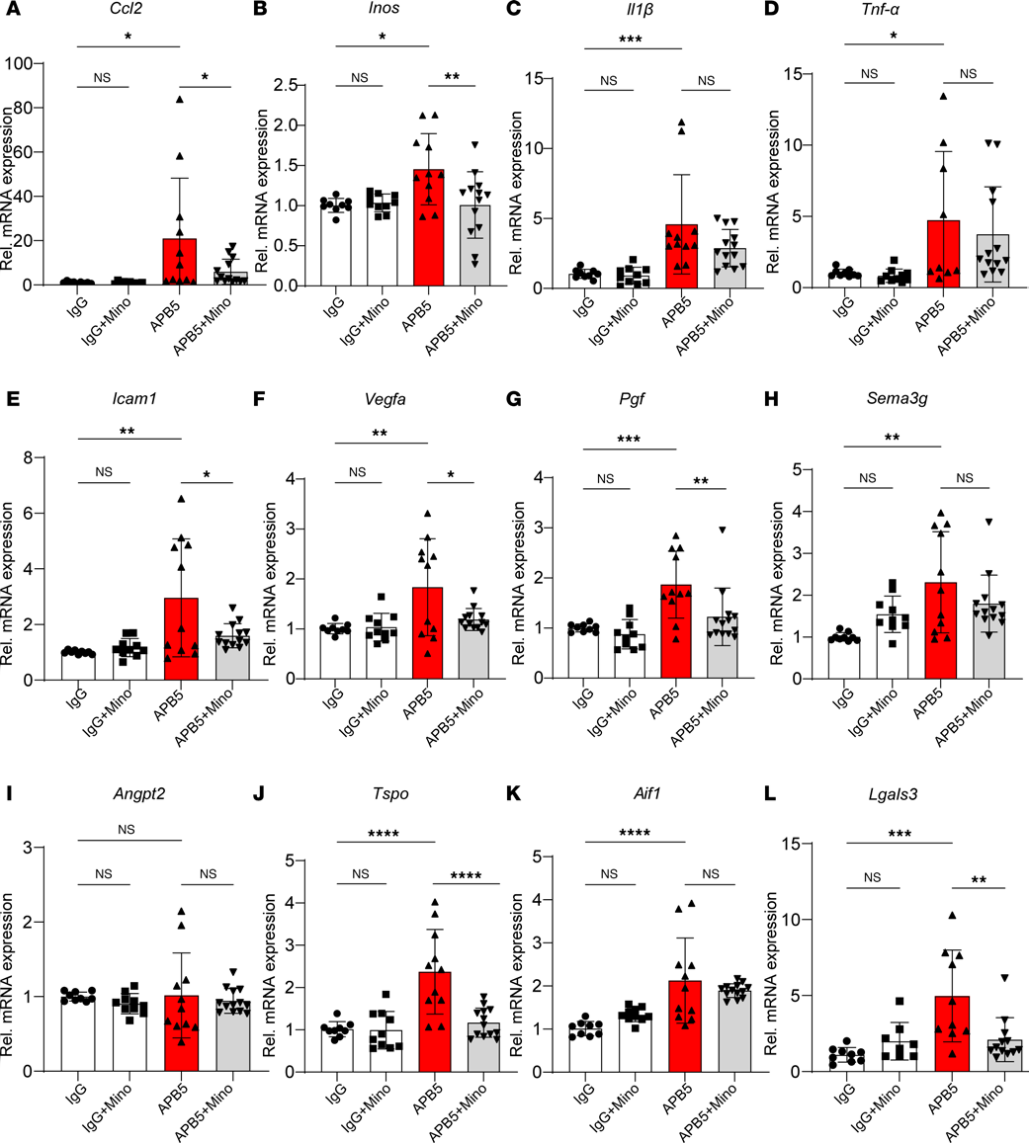
qPCR analyses of inflammatory and proangiogenic factors in P10 retinas. (**A**–**D**) APB5 induced the expression of proinflammatory genes. *Ccl2*, *Inos*, *Il1β*, and *Tnfa*. Minocycline suppressed the mRNA levels of *Ccl2* and *Inos* but not *Il1β* or *Tnf-*α. (**E**–**I**) Proangiogenic genes; *Icam1*, *Vegfa*, *Pgf*, and *Sema3g* were upregulated in APB5 retinas, but the mRNA level of *Angpt2* was not significantly different from control groups. (**J**–**L**) The mRNA levels for markers of activated microglia. *Tspo*, *Aif1*, and *Lgals3* were significantly upregulated in APB5 retinas and downregulated by minocycline with the exception of *Aif1* (*n* = 9–13). *P < 0.05, **P < 0.01, ***P < 0.001, ****P < 0.0001. One-way ANOVA.

Besides the morphological transformation, microglia reactivity entails the secretion of a repertoire of inflammatory factors in the pathogenesis of DR (28). Since microglia activation was prominent in retinas of APB5-treated mice at P10, we sought to investigate the expression of disease-relevant proinflammatory and angiogenic factors and their response to minocycline. The mRNA levels of chemokines and cytokines such as *Ccl2*, *Inos*, *Il1b*, and *Tnf-*α (Figure 3, A–D) and proangiogenic factors, *Icam1*, *Vegfa*, *Pgf*, and *Sema3g* were elevated in APB5 retinas (Figure 3, E–H) in contrast to control retinas. However, the transcript levels of *Angpt2* did not differ significantly among treatment groups (Figure 3I). Also, key transcripts that denote activated microglia including *Tspo*, *Aif1*, and *Lgals3* were significantly upregulated in the APB5 retinas (Figure 3, J–L). Treatment with minocycline significantly reduced the transcript levels of *Ccl2*, *Inos*, *Icam1*, *Vegfa*, *Pgf*, *Tspo*, and *Lgals3*, whereas the levels of *Il1b*, *Tnf-*α, *Sema3g*, and *Aif1* did not differ appreciably between the APB5 and minocycline-treated APB5 mice. Since both VEGF and PIGF are potent angiogenic factors that aggravate disease outcome during the progression of retinal vascular pathologies, we sought to investigate their cellular sources within the retina by RNAscope in situ hybridization (ISH) on retinal sections. Our findings show that *Vegf* mRNA was expressed within the INL and in the GCL of minocycline-treated groups (Supplemental Figure 2, A and B), whereas *Pgf* mRNA was distributed across the GCL and INL (Supplemental Figure 1, C and D). Notably, the signal intensity of both the *Vegf* and *Pgf* mRNAs within the INL was higher in the APB5-treated retinas when compared controls and APB5 retinas treated with minocycline. This observation implies that VEGF and PIGF are secreted by cells within the INL and GCL, suggesting a role of Müller cells in DR pathogenesis. Notably, minocycline-treated APB5 retinas exhibited a low signal intensity of both *Vegf* and *Pgf* mRNAs. Taken together, these findings suggest that, at a molecular level, minocycline functions by resolving microglia-related inflammatory responses and limits harmful angiogenic responses in pericyte depletion retinopathy.

### Sustained inflammation after postnatal pericyte loss causes visual deficits in adult mice.

**Figure 4:**
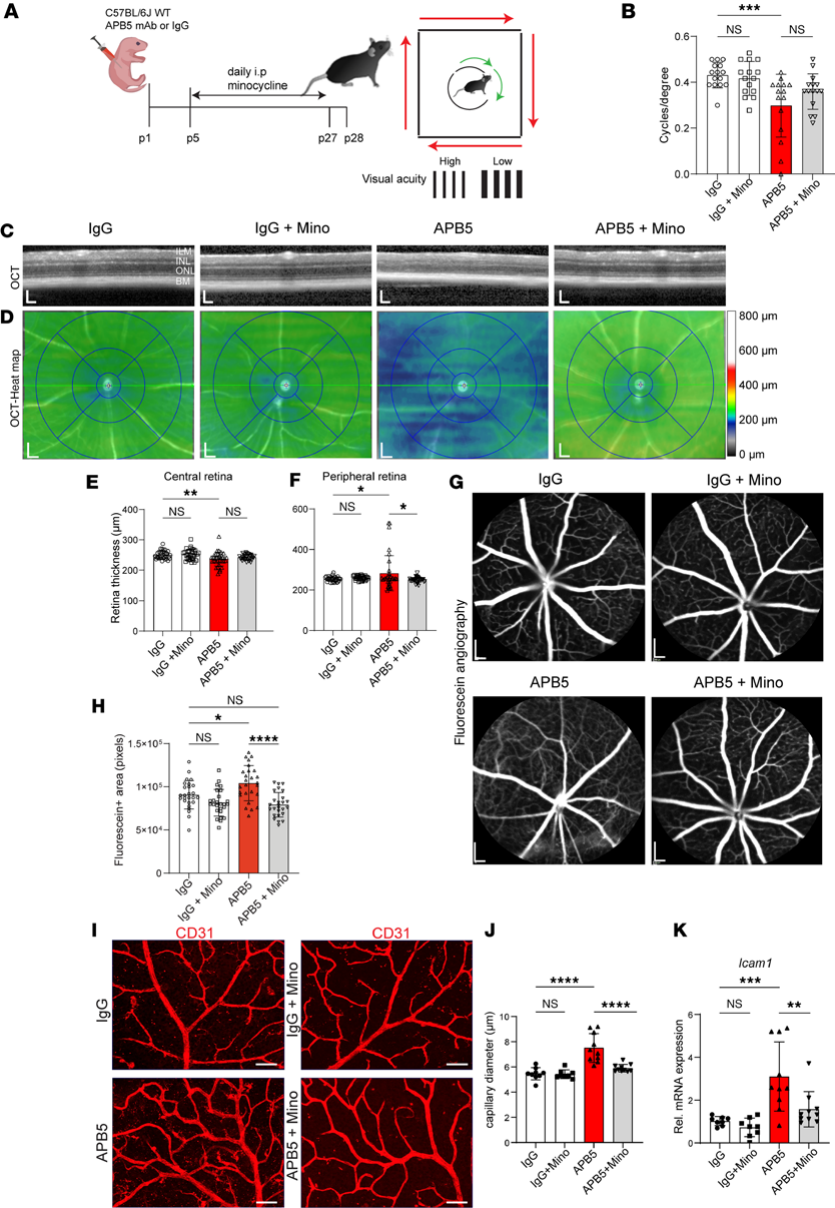
APB5-induced retinopathy triggers changes in retinal visual function and structural integrity in 4-week-old mice. (**A**) Schematic representation of treatment paradigm and analysis of visual acuity testing with the OptoDrum. (**B**) Quantification of scores of contrast sensitivity (cycles per degree) as a test of visual acuity (*n* = 15 mice). (**C** and **D**) Representative images of automated SD-OCT scans of mice retinas (**C**) and corresponding SD-OCT heatmaps (**D**); green indicates the average retina thickness in adult mice while blue indicates retina thinning. (**E** and **F**) Quantification of retina thickness within the central (**E**) and peripheral regions (**F**), which correspond to 3 mm and 6 mm, respectively, from the optic nerve head (*n* = 34–39 eyes). (**G** and **H**) Representative images of fluorescein angiography showing vessel permeability in the retina (*n* = 26 eyes per group). (**I**) IHC for CD31 on retinal capillary plexus. (**J** and **K**) Analyses of retinal capillary diameter (*n* = 8–10 retinas) and quantitative analysis of the transcript levels of *Icam1* in whole retina lysates (*n* = 8–10 retinas). *P < 0.05, **P < 0.01, ***P < 0.001, ****P < 0.0001. Data show mean ± SD. Scale bar: 200 μm (**G**), 50 μm (**H**). One-way ANOVA.

In 4-week-old APB5-treated mice, the visual acuity as analyzed with an OptoDrum was reduced when compared with control mice (Figure 4A). Notably, APB5-treated mice showed a decrease in visual acuity (mean of 0.32 cycles/degree) compared with the control group (mean of 0.41 cycles/degree). In addition, minocycline slightly enhanced the visual acuity of APB5 mice (mean of 0.38 cycles/degree), but this change was not significantly different compared with the APB5-only group (Figure 4B). Next, we analyzed the structural integrity of the neural retina and vasculature in vivo by spectral-domain optical coherence tomography (SD-OCT) and fluorescein angiography (FA). The SD-OCT scans showed preserved retinal structure in control mice, but alteration of the retina was observed in the APB5-treated mice, which was also manifested by the blue color of the corresponding OCT heatmap (Figure 4, C and D). Automated quantification of the central (3 mm region) and peripheral (6 mm region) retina thickness using the HEYEX software of SD-OCT showed that minocycline preserved retinal structural integrity and limited retina thinning at the peripheral region but not the central region (Figure 4, E and F). Moreover, FA showed fluorescein leakage in retinal capillaries of APB5 mice (Figure 4G), which is consistent with the clinical observation in patients with DR, but this leakage was diminished in minocycline-treated mice, thereby indicating preservation of retinal capillaries from damage (Figure 4H). Next, we examined the patterning of retinal vasculature in 4-week-old mice. In the superficial plexus, we observed dilated capillaries in the APB5 retinas, but no significant changes were detected in the vascular density of the superficial vasculature (Figure 4I). Treatment with minocycline rescued capillary diameter and decreased the levels of ICAM1 in the APB5-treated mice (Figure 4, J and K).

### Minocycline dampens microglia activation in the mature mouse retina.

**Figure 5:**
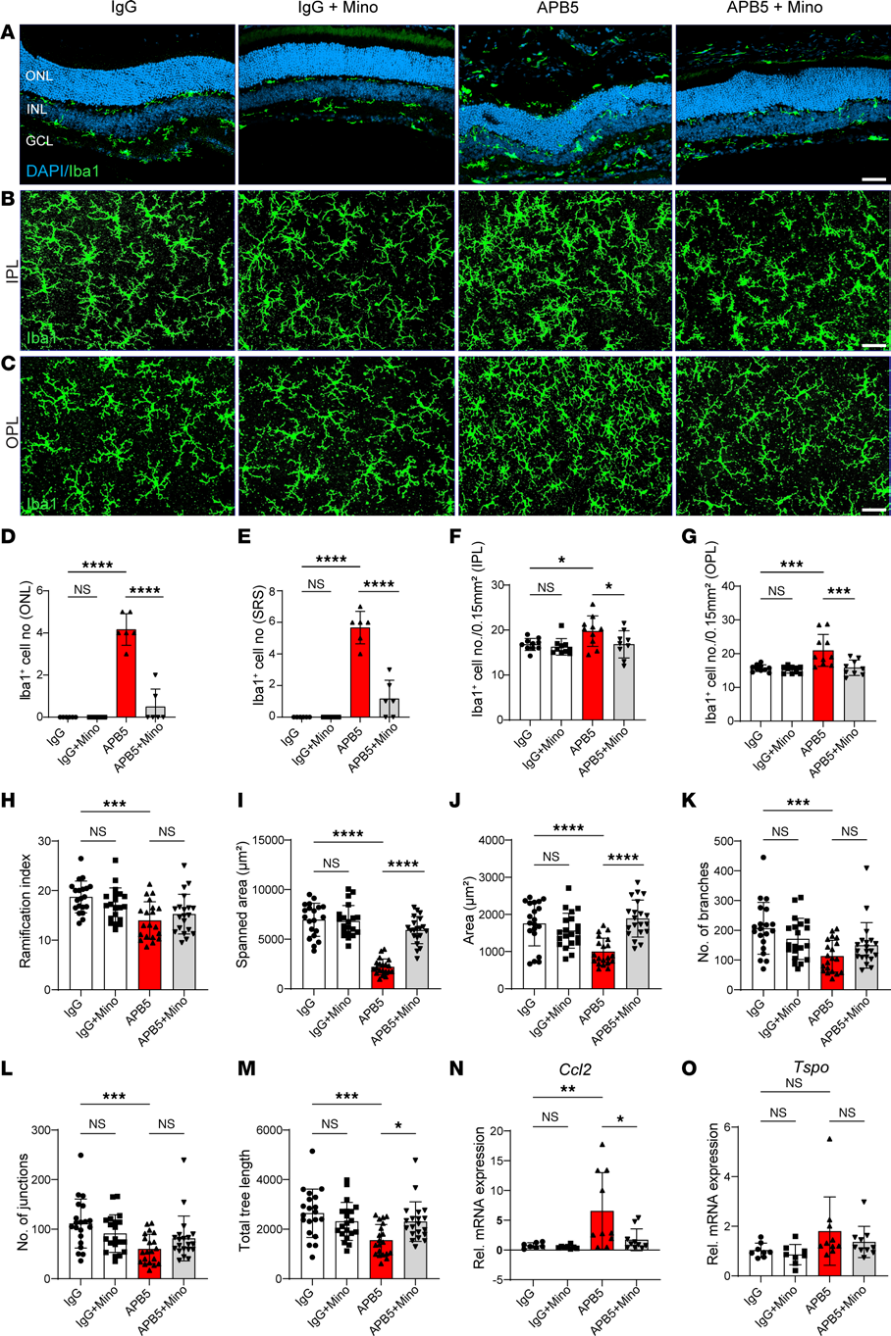
Minocycline limits APB5-triggered microglia reactivity in mature retinas. (**A**) IHC for Iba1 on retina cross-sections showing microglia localization and migration across retinal layers of APB5-treated mice and modulation by minocycline. (**B** and **C**) IHC for Iba1 on retinal whole mounts showing microglia morphology in the IPL (**B**) and OPL (**C**). (**D** and **E**) Quantification of Iba1^+^ cells within the ONL (**D**) and SRS (**E**) in retina sections (*n* = 6 retinas). (**F** and **G**) Quantification of Iba1^+^ cells within an area of 0.15mm^2^ in the IPL (**F**) and OPL (**G**) (*n* = 9–10 retinas). (**H**–**M**) Analysis of the morphometric attributes of microglia within the OPL, ramification index (**H**), spanned area (**I**), area (**J**), number of branches (**K**), number of junctions (**L**), and tree length (**M**) (*n* = 20 Iba1^+^ cells across all experimental groups). (**N** and **O**) qPCR analyses of the mRNA expression levels of chemokine CCL2 (**N**) and translocator protein (18Kda) TSPO (**O**) (*n* = 8–10 retinas). *P < 0.05, **P < 0.01, ***P < 0.001, ****P < 0.0001. Data show mean ± SD. One-way ANOVA.

Besides the vascular patterning, we investigated the phenotype of microglia in mature retinas. Consistent with the observation in P10 retinas, APB5 induced and sustained the activation of microglia within the IPL and OPL of mature retinas. Notably, microglia portrayed a migratory phenotype where they migrated across the ONL into the SRS (Figure 5, A–C). Prolonged treatment with minocycline reduced the migratory capacity of microglia and, hence, the number of Iba1^+^ cells in the ONL, SRS, and in the IPL as well as OPL (Figure 5, D–G). Next, we analyzed the key morphometric attributes of microglia in the OPL as an indicator of microglia activation. Here, we chose to analyze Iba1^+^ cells in the OPL because we recorded a high degree of visible microglia activation in this region. Microglia in APB5 retinas had a significantly reduced ramification index, spanned area, number of branches, number of junctions, and tree length (Figure 5, H–M) than did IgG control mice. Although treatment with minocycline significantly increased the spanned area and tree length of microglia in APB5 retinas, it did not restore the ramification index, number of branches and junctions to the homeostatic state. Further analyses of retinal lysates detected a significant upregulation of CCL2, an important chemokine for recruitment of phagocytes, but the levels of TSPO did not differ significantly among the experimental groups (Figure 5, N and O). Thus, minocycline reduced the recruitment of microglia, likely via downregulating of CCL2 in APB5 retinas. Since astrocytic stress is also a common feature of retinal pathologies, we investigated the expression GFAP in astrocytes and Müller glia in APB5 mice with or without minocycline treatment. IHC clearly revealed the presence of GFAP stress fibers in retinal Müller cells of APB5-treated mice, which was abrogated in minocycline-treated retinas (Supplemental Figure 2, E–G).

### Anti-PDGFRβ mAb and minocycline induced global transcriptomic changes in the retina.

**Figure 6:**
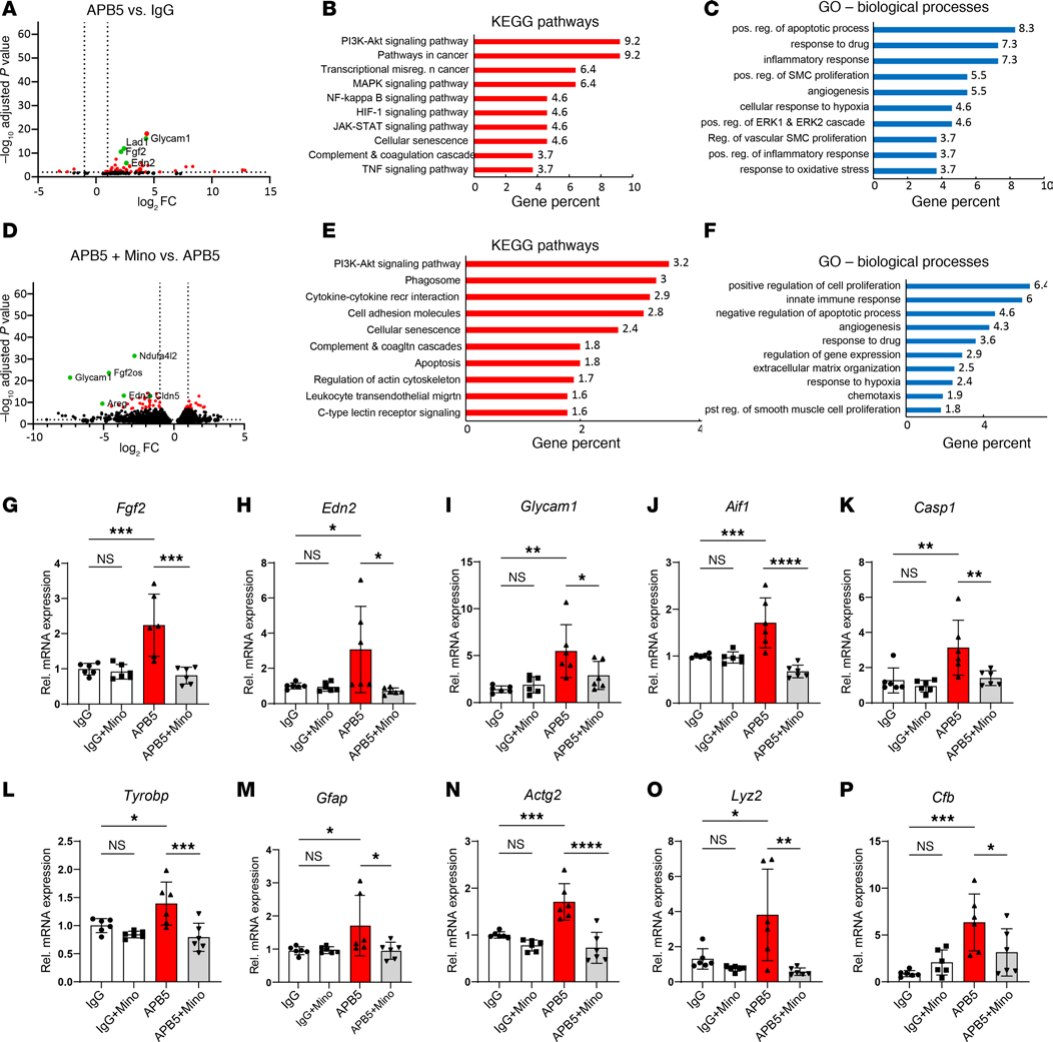
APB5 and minocycline exert global changes in retinal transcriptome of 4-week-old retinas. (**A**) A volcano plot showing comparison of DEGs between IgG and APB5 retinas. (**B**) KEGG pathway analysis showing the top 10 enriched pathways in APB5 retinas. (**C**) GO domain biological processes showing key processes enriched by APB5. (**D**) A volcano plot showing comparison of DEGs between APB5 and APB5 with minocycline retinas. (**E**) KEGG pathway analysis shows the top 10 pathways enriched by minocycline. (**F**) GO domain biological processes showing key processes enriched by minocycline in APB5 retinas. The green dots on the volcano plots label some of the DEGs. The set cut-off for the DEGs shown on the volcano plot is log_2_ FC of ± 1 and FDR of < 0.05; *n* = 3 mice in each group. (**G**–**P**) Verification of DEGs by qPCR in whole retinal lysates; *FgF2*, *Edn2*, *Glycam1*, *Aif1*, *Casp1*, *Tyrobp*, *Gfap*, *Actg2*, *Lyz2*, and *Cfb*. The mRNA levels of these factors were significantly reduced in retinas of minocycline treated mice (*n* = 6 retinas). *P < 0.05, **P < 0.01, ***P < 0.001, ****P < 0.0001. Data show mean ± SD. One-way ANOVA.

We then investigated the effect of APB5 and minocycline on the retinal transcriptome by performing RNA-Seq of retinal tissues of 4 weeks of age. Comparative analyses of the gene expression profile between the APB5 and IgG retinas identified 116 differentially expressed genes (DEGs), out of which 102 were upregulated, whereas 5 were downregulated at a cut-off set to log_2_ fold change ± 1 and *P* ≤ 0.05. Among the top DEGs were genes related to complement system such as *C4b*, *Cfi*, *C3*, and *C1qa* and inflammation-related genes including *Lyz2*, *Glycam1*, *Fgf2*, *Edn2*, and *Icam1*, some of which are represented on the volcano plot (Figure 6A). To identify pathway enrichment in these retinas, we performed gene set enrichment analyses of all DEGs using the Kyoto Encyclopedia of Genes and Genomes (KEGG) and Gene Ontology (GO) database. The top enriched KEGG pathways in APB5 retinas were involved in inflammation and diseases such as cancer, HIF-1 signaling, complement and coagulation cascades, and cellular senescence that mirror the pathogenesis of proliferative DR (Figure 6B). Furthermore, the GO domain biological processes revealed inflammation as well as hypoxic and vascular remodeling among the top APB5-induced processes (Figure 6C). Similarly, we investigated the effects of minocycline relative to APB5 on the retinal transcriptome. Differential gene expression analyses identified 981 downregulated and 304 upregulated genes at a cut-off set to log_2_ fold change ± 1 and *P* ≤ 0.05. Among the DEGs upregulated in the APB5 retinas, minocycline downregulated *Fgf2*, *Glycam1*, *Ndufa4l2*, and *Edn2* as illustrated in the volcano plot (Figure 6D). Next, we performed gene set enrichment analysis of all the 1,285 DEGs (both upregulated and downregulated) to identify enriched pathways. The KEGG analysis identified inflammation-associated pathways (P13k/Akt signaling, phagosome, cytokine-cytokine interaction, complement and coagulation cascades, c-type lectin receptor signaling), vascular remodeling pathways (cell-adhesion molecules, regulation of actin cytoskeleton, leukocyte transendothelial migration), and cell death and senescence pathways as key pathways regulated by minocycline (Figure 6E). The GO domain biological processes identified angiogenesis, response to hypoxia, regulation of genes expression, and response to drug as pathways sustained by minocycline (Figure 6F).

Consequently, we performed gene expression analyses by qPCR to validate the RNA-Seq transcriptome data with disease-relevant marker genes. We detected a strong upregulation of mRNA levels of *Fgf2*, *Edn2*, *Glycam1*, *Aif1*, *Casp-1*, *Tyrobp*, *Gfap*, *Actg2*, *Lyz2*, and *Cfb* in the APB5-treated retina, which were significantly reduced in minocycline treated retinas (Figure 6, G–P). The expression of genes encoding angiogenic factors (*Fgf2* and *Edn2*), mediators of inflammatory leukocyte trafficking (*Glycam-1*), and immune cell inflammatory factors (*Aif1*, *Casp-1*, *Lyz2*, and *Tyrobp*) and their suppression by minocycline aptly reflects the role of inflammatory responses in disease progression and the immunomodulatory effect of minocycline.

Although FGF2 is an angiogenic factor with secretion dynamics that remain unresolved, it is expressed by necroptotic microglia in hypoxia-induced retinopathy (29). Therefore, we sought to investigate whether APB5-induced retinopathy triggered the expression of FGF2 by microglia in the IPL, OPL, and SRS. Our findings showed a minimal coexpression of FGF2 and Iba1 in cells within the IPL and OPL of the control groups, whereas a high coexpression was observed in the APB5 groups (Supplemental Figure 3, A–C). Quantification of FGF2^+^Iba1^+^ cells established that the APB5-treated retinas had a high number of these cells, which was reduced in the minocycline-treated group (Supplemental Figure 3D).

Surprisingly, *Vegf* was not among the DEGs in the APB5 retinas relative to control retinas. Our ISH analyses detected *Vegf* mRNA in the INL of retinal sections but without notable difference in its distribution across treatment groups (Supplemental Figure 3, E–G), thereby validating the results of RNA-Seq. In addition, the analysis of gene counts of *Vegf* mRNA showed no significant differences in the expression profile of Vegf isoforms (Supplemental Figure 3, H–K).

As microglia reactivity was prevalent in the pericyte-depleted retinas by 4 weeks of age, we sought to investigate the progression of disease by depleting CSF-1R–expressing cells. Using this approach, we aimed at studying the effect of systemic macrophage depletion in the retina. Here, we show that CSF-1R inhibition by PLX3397 effectively depleted Iba1^+^ cells within the retina but without diminishing GFAP expression (Supplemental Figure 4, A–C). Although the depletion of CSF-1R–expressing cells reduced vascular permeability as a measure of fluorescein intensity (Supplemental Figure 4, D and E), the loss of these cells was not sufficient to reverse capillary APB5-induced capillary dilation (Supplemental Figure 4F). Further analysis of the mRNA levels of markers of microglia reactivity and angiogenesis revealed that pharmacological depletion of CSF-1R^+^ cells remarkably reduced the expression of Aif1, CCl2, and Icam1 but not Pgf (Supplemental Figure 4, G–J). Collectively, depletion of microglia and other mononuclear phagocytes, including infiltrating peripheral monocytes and leukocytes, modulates inflammatory gene expression and reduces vascular permeability but without absolute rescue of capillary size.

### Endothelial deletion of PDGFB impairs BRB integrity.

**Figure 7:**
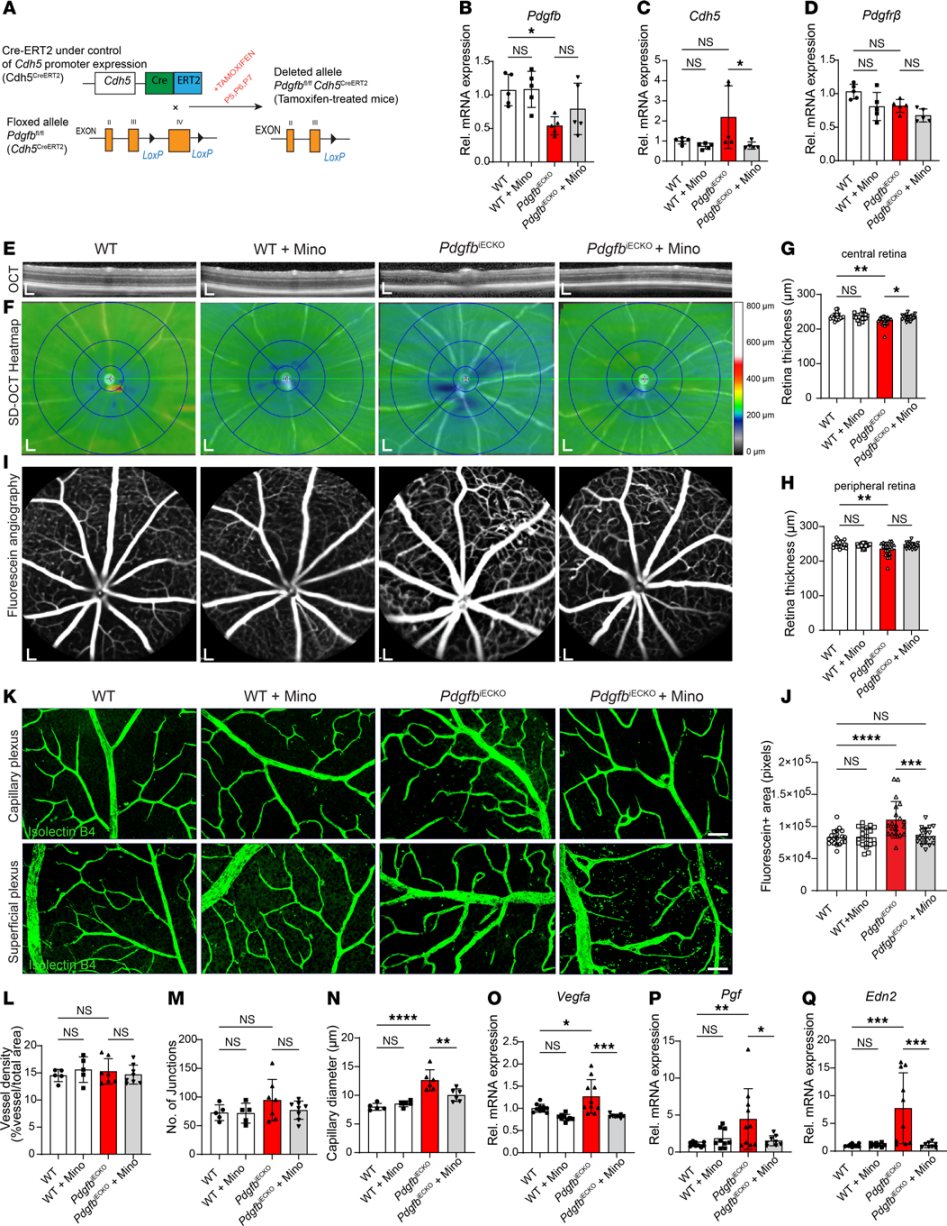
Depletion of PDGFB in retinal vascular endothelium. (**A**) Schematic representation of tamoxifen inducible depletion of PDGFB in retinal endothelium under the control of Ve-Cdh5 promoter. (**B**–**D**) qPCR analysis of mRNA expression levels of *Pdgfb* (**B**), *Cdh5* (**C**), and *Pdgfrβ* (**D**). (**E** and **F**) Representative images of automated SD-OCT scans of mice retinas (**E**) and the corresponding SD-OCT heat maps (**F**). (**G** and **H**) Quantification of retina thickness within the central (**G**) and peripheral regions (**H**), corresponding to 3 mm and 6 mm, respectively, from the optic nerve head (*n* = 20 eyes per group). (**I** and **J**) Representative images of fluorescein angiography showing vessel engorgement and permeability (**J**, *n* = 20 eye per group). (**K**–**N**) IHC with Isolectin B4 showing vascular network in the capillary and superficial plexus (**K**), and analyses of vascular density (**L**), number of junctions (**M**) (*n* = 5–8 retinas), and capillary diameters (**N**) (*n* = 5–6 retinas). (**O**–**Q**) qPCR analyses of angiogenic factors *Vegfa* (**O**), *Pgf* (**P**), and *Edn2* (**Q**) in whole retina lysates of 4-week-old mice (*n* = 8–10 retinas). Scale bar: 200 μm (**E**, **F**, and **I**) and 50 μm (**K**). *P < 0.05, **P < 0.01, ***P < 0.001, ****P < 0.0001. Data show mean ± SD. One-way ANOVA.

The retention of sufficient PDGFB in the vascular endothelium is indispensable for proper recruitment and investment of pericytes to ECs (30). A significant reduction of pericyte density in retinal vasculature during development leads to a phenotype reminiscent of human DR in mice (31). In order to gain deeper insights into pathogenesis of DR upon genetic attenuation of PDGFB/PDGFRβ signaling, we depleted PDGFB in retinal endothelium in a tamoxifen-inducible manner by crossing the *Pdgfb*^fl/fl^ (31) with the VE-Cadherin-Cre^ERT2^ (32) to generate conditional EC PDGFB-KO mice, *Pdgfb*^iECKO^ (Figure 7A). Quantitative PCR (qPCR) analyses of *Pdgfb* transcripts in whole retina lysates revealed that *Pdgfb* mRNA levels were significantly reduced by almost 50% in the *Pdgfb*^iECKO^ retinas compared with WT mice (Figure 7B). The transcript levels of *Cdh5* and *Pdgfrβ* did not vary among the groups (Figure 7, C and D).

Next, we investigated the retinal structure and vessel integrity in vivo in the 4-week-old mice. SD-OCT analyses revealed thinning of the retina in the central and peripheral regions of the *Pdgfb*^iECKO^ mice (Figure 7, E and F). Treatment of *Pdgfb*^iECKO^ mice with minocycline restored retinal thickness of the central retina but not the peripheral retina (Figure 7, G and H). Vascular permeability as measured by FA showed that the enlarged capillaries visible in *Pdgfb*^iECKO^ retinas were diminished in minocycline-treated mice (Figure 7, I and J). IHC with Isolectin B4 on retinal whole mounts, and subsequent analysis of the superficial vasculature revealed no significant differences in the vascular density or branching index but did reveal significant differences in capillary diameter between WT and *Pdgfb*^iECKO^ mice retinas; these differences marked by dilated capillaries were rescued with minocycline (Figure 7, K–N). Subsequent analyses by qRT-PCR showed that the levels of key EC inflammatory and vascular destabilization factors VEGF, PIGF, and EDN2 were significantly upregulated in PDGFB-depleted retinas, but significantly reduced in retinas of minocycline-treated mice (Figure 7, O–Q). Taken together, these findings imply that minocycline exerted a modulatory effect on capillary ECs either directly or indirectly by dampening the proangiogenic responses of ECs to preserve capillary structure.

### Loss of PDGFB in ECs leads to inter- and intraindividual variation in disease outcome.

Even though we used an inducible genetic method to deplete PDGFB in retinal endothelium, we observed intraindividual variation in the disease outcome. For instance, FA showed retinal vascular engorgement in one eye but not the other eye in the same PDGFB-depleted mouse (Supplemental Figure 5). In addition, angiographic analyses of *Pdgfb*^iECKO^ retinas revealed that the vascular abnormalities and leakage were more likely to occur in both eyes of males than females (Supplemental Table 1). Among the male mice, the likelihood of the phenotype occurring in the left and right eye was 80% and 50%, respectively. In retinas of *Pdgfb*^iECKO^ female mice, the likelihood of the vascular phenotype occurring in the left and right eye was 50% and 50%, respectively. Previously, Enge and colleagues reported the intra- and interindividual variation in disease phenotype in endothelium-restricted PDGFB loss in the CNS but with a focus on pericyte coverage (31). Moreover, the intraindividual variation in disease progression in the PDGFB-depleted retinas is reminiscent of the clinical progression of human DR, where retinal microvasculopathy first manifests in one of the eyes of a DR patient during the progression of the disease.

### Minocycline limits adverse microglia activation in PDGFB-depleted retinas.

**Figure 8:**
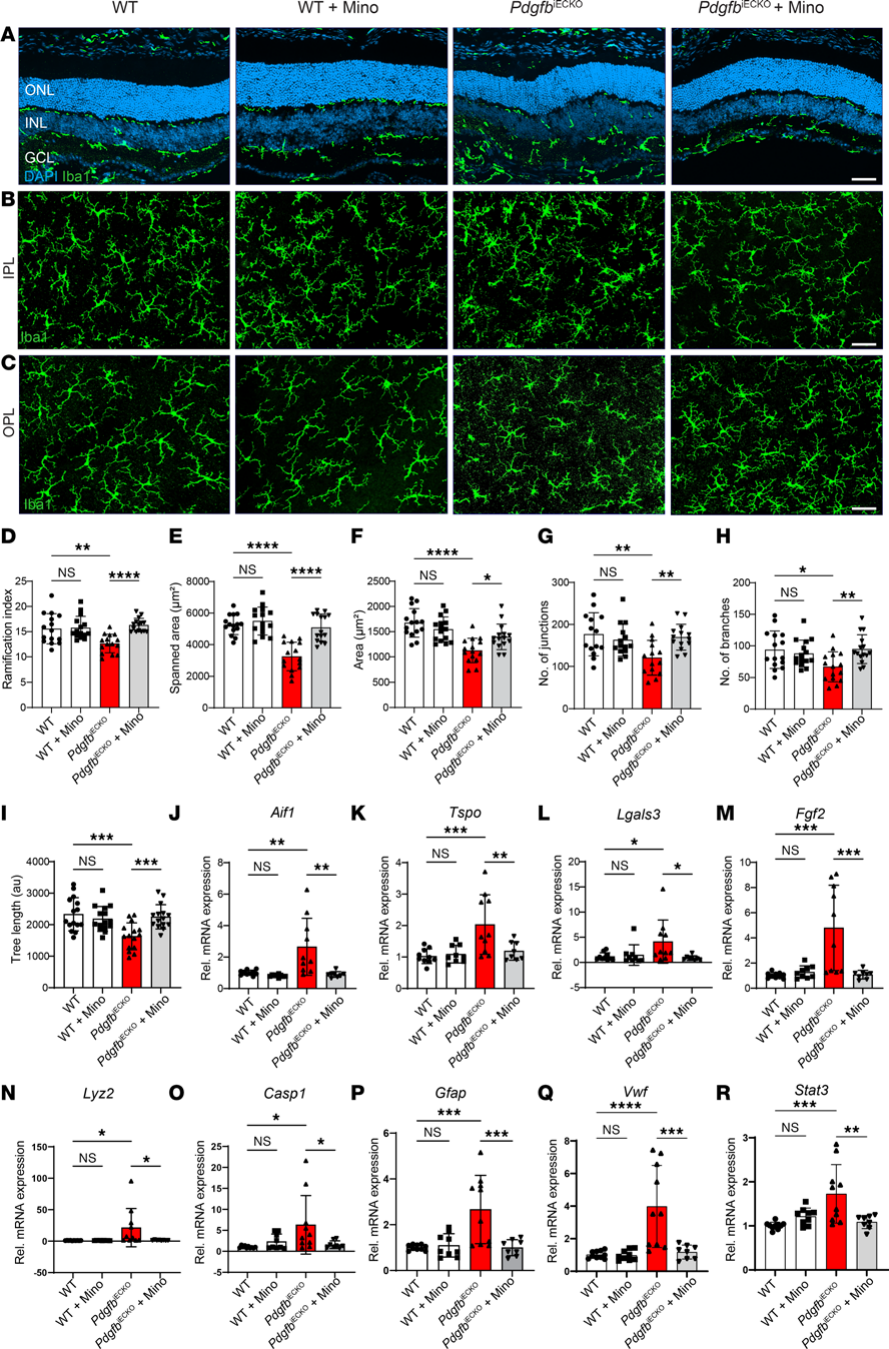
Endothelium-specific conditional deletion of *Pdgfb* triggers microglia reactivity in adult mice retina. (**A**) IHC for Iba1 on retina cross sections showing microglia localization and migration across retinal layers of Pdgfb-depleted and WT retinas with or without minocycline. (**B** and **C**) IHC for Iba1 on retinal whole mounts showing microglia morphology in the IPL (**B**) and OPL (**C**). (**D**–**I**) Quantification of morphometric attributes of Iba1^+^ microglia within the OPL, ramification index (**D**), spanned area (**E**), area (**F**), number of branches (**G**), number of junctions (**H**), and tree length (**I**) (*n* = 15 Iba1^+^ cells across all experimental groups). (**J**–**R**) qPCR analyses of the mRNA expression levels of inflammatory factors, *Aif1* (**J**), *Tspo* (**K**), *Lgals3* (**L**), *Fgf2* (**M**), *Lyz2* (**N**), *Casp1* (**O**), *Gfap* (**P**), *Vwf* (**Q**), and *Stat3* (**R**) (*n* = 8–10 retinas). Scale bar: 50 μm. *P < 0.05, **P < 0.01, ***P < 0.001, ****P < 0.0001. Data show mean ± SD. One-way ANOVA.

We next asked whether impaired vascular patterning in PDGFB-depleted retinas was associated with microglia activation. To answer this question, we performed IHC with Iba1 on retina sections and whole mounts. In the WT mice, with or without minocycline, Iba1^+^ cells occupied the IPL and OPL, where they exhibited a homeostatic ramified morphology. In contrast, Iba1^+^ cells in the PDGFB-depleted retinas exhibited an activated morphology indicated by clamping together in the OPL, which was reversed in minocycline-treated retinas (Figure 8, A–C). In contrast to WT and minocycline-treated mice, microglia within the OPL of PDGFB-depleted retinas portrayed a decrease in ramification index, spanned area, area, number of junctions, number of branches, and tree length as analyzed with micromorphometry (Figure 8, D–I).

Minocycline treatment also downregulated several transcripts associated with activated microglia including *Aif1*, *Tspo*, *Lgals3*, *Fgf2*, and *Lyz2* in the PDGFB-depleted retinas (Figure 8, J–N). Furthermore, minocycline downregulated activators of cellular inflammation and EC dissociation such as *Casp1*, *Gfap*, *Vwf*, and *Stat3* in the diseased retinas (Figure 8, O–R). Thus, inhibiting microglia reactivity with minocycline not only suppressed secretion of inflammatory factors but also downregulated *Fgf*2, *Stat*3, and *Vwf* transcripts, which sustain inflammation of ECs and breakdown of tight junctions in retinal endothelium.

### Depletion of PDGFB in retinal ECs leads to secretion of proangiogenic factors and Müller cell gliosis.

Since the expression of FGF2 and VEGF was abundant in PDGFB-depleted retinas, we investigated their cellular sources in situ using RNAscope hybridization technology. We detected an intense signal of *Vegf* mRNA within the cell bodies of Müller glia, marked by glutamine synthetase, within the INL of PDGFB-depleted retinas, which was suppressed in minocycline-treated retinas (Supplemental Figure 6, A–D). In the retinas of WT mice, *Fgf2* mRNA was localized within the INL but was detected in the ONL of PDGFB-depleted mice (Supplemental Figure 6E). In addition, *Fgf2* mRNA did not colocalize with *Aif1* mRNA or Iba1 (Supplemental Figure 6, F–H).

Furthermore, we investigated reactive gliosis — specifically, whether Müller cells exhibited increased expression of GFAP in the PDGFB-depleted retinas. IHC with GFAP and glutamine synthetase revealed that loss of PDGFB in retinal ECs was associated with reactive gliosis of Müller cells, which was suppressed by minocycline (Supplemental Figure 7, A–C). Taken together, minocycline limits Müller cell gliosis within the retina, thereby modulating disease progression and protecting the retina from glia reactivity.

## Discussion

The destabilization of the structural components of the iBRB during postnatal life in mice leads to the breakdown of BRB and leakage of blood-derived components into the retina, and it triggers chronic inflammation (18, 19), which are key pathological processes in the etiology of DR. Furthermore, the dilation of retinal vessels with concomitant increase in superficial vascular density is a consequence of loss of integrity of tight-junction proteins in pericyte-free retinas.

In the present study, we show that treatment with minocycline preserved BRB components, suggesting that the therapeutic intervention promoted mitotic activity of ECs, reconstitution of tight junctions, and sprouting of ECs into the deep vascular plexus. In addition, minocycline protected ECs from cellular damage by downregulating key drivers of pathological angiogenesis, including PIGF, VEGF, ICAM-1, and SEMA3G in APB5 retinas. In patients with DR, elevated levels of SEMA3G, PIGF, VEGF, and ICAM in the vitreous and aqueous humor correlate with progression and severity of PDR (33–35). Indeed, dysregulated secretion of these angiogenic factors by the endothelium sensitizes ECs toward an inflammatory loop between endothelium and immune cells leading to chronic inflammation (18).

The antiinflammatory effect of minocycline at the early stage of pericyte-depletion retinopathy was corroborated by the downregulation of CCL2, iNOS, TSPO, and LGALS3 in minocycline-treated APB5 retinas at P10. The reduction in CCL2, a potent chemokine involved in macrophage recruitment, TSPO, and LGALS3 that we have previously shown to be highly expressed in activated microglia (36, 37) implies that minocycline dampened microglia recruitment and prolonged activation in the APB5-treated retinas at P10. In partial alignment with our studies, previous studies in the APB5 retinas detected infiltration of perivascular macrophages at P6 and P10 (18, 27), possibly driven by CCL2 activity. Taken together, these findings suggest that minocycline prevents damage of the iBRB structural components by suppressing excessive secretion of harmful angiogenic factors and limits microglia inflammatory responses in the postnatal murine retina.

Although minocycline preserved photoreceptors in the APB5 model of pericyte depletion, the effect was not sufficient to restore visual acuity in adult mice. Since visual function also relies on the signaling activity of retinal neurons, our findings imply that the recovery of photoreceptor density in minocycline-treated mice may occur prior to restoration of the complete function of bipolar and amacrine cells that generate action potentials to transmit electric signals across the retina. Minocycline reduced microglia migration across the nuclear layers and reduced the increased recruitment of Iba1^+^ cells within the IPL and OPL of APB5 retinas. Although the precise molecular target of minocycline in this pathology is yet to be identified, the effect exerted on microglia was achieved through targeting AIF1, LYZ2, CCL2, and FGF2 in the retina, thereby affecting the retinal secretome and restoring the homeostatic phenotype of microglia. The present findings support previous studies including our own that showed minocycline inhibited microgliosis and photoreceptor loss in mouse models of age-related retinal degeneration and P23H-1 (rhodopsin mutation) rat model of retinal degeneration (21, 22, 38, 39). Similarly, minocycline was reported to suppress microglia activation and release of cytokines in a rat model of Streptozotocin-induced diabetes (25). While the STZ models recapitulate systemic hyperglycemia, a key feature in the development of DR, they fall short of fully recapitulating the ocular phenotype associated with DR, especially the complex vascular and neuronal changes associated with pericyte loss (40, 41). Using the model of pericyte-depletion retinopathy, we have advanced the existing knowledge of the modulatory effect of minocycline on global retina inflammation by targeting both proinflammatory and proangiogenic factors.

Furthermore, characterization of the microglia secretome showed that the angiogenic factor FGF2 was highly expressed by Iba1^+^ microglia in the APB5 retinas, but its mRNA, which was reserved within the INL of WT mice, FGF2 mRNA translocated to the ONL in PDGFB-depleted retinas. Although the secretion dynamics of FGF2 in the retina remain unresolved, FGF2 expressing retinal microglia were found to drive pathological angiogenesis in retinopathy via either hypoxia-triggered necroptosis or lactylation in microglia (29, 42). It remains unclear whether neutralization or genetic targeting of FGF2 would offer improved therapeutic benefits, such as protecting the retina from inflammation and vascular dysfunction, or enhancing visual acuity. The clinical progression of PDR is mostly associated with VEGF for which treatment is designed. However, 40%–60% of patients with DR fail to respond to anti-VEGF therapy (7, 43), raising the need for alternative therapies. The identification of FGF2, as dually expressed by microglia and vasculature in APB5 retinopathy, makes it a potential immunomodulatory target. In the APB5 model, the recapitulation of the human DME, particularly thickening of the retina, may be dependent on the amount of APB5 mAb administered. In the present study, 30 μg was adequate to impair vascularization and trigger photoreceptor loss in adult mice. Other studies have shown that a higher dose of APB5 reproduces the phenotype of human DME in mice (44), which is an irreversible stage.

Since chronically activated microglia are key contributors to retinal pathologies, we established that depletion of all CSF-1R–expressing macrophages with PLX3397 protected the retina from elevated inflammation and vascular leakage although without complete reversal of dilated capillaries. This suggests that microglia may play an intrinsic role in the restoration of vascular structure, an observation that warrants more study. We have previously shown that PLX3397 ameliorated expression of proinflammatory cytokines in the retinas of light-exposed mice but without a rescue of photoreceptor demise (45).

In adult mice, minocycline preserved BRB integrity by reducing fluorescein leakage and capillary engorgement in both APB5 and *Pdgfb*^iECKO^ mouse retinas. The failure of the BRB is a major pathological feature of DR often accompanied by thinning of the ONL and loss of vision (3, 7). The protective effect of minocycline on the BRB was corroborated with decreased levels of ICAM1, EDN2, and FGF2 in both models as well as reduced levels of VEGFA, VWF, and PIGF in PDGFB-depleted retinas. In the pericyte and PDFGB-depletion microvasculopathy, we highlight the role of EDN2, a potent vasoactive factor that overrides the effect of homeostatic regulators of angiogenesis and causes vascular alterations in the diabetic retina (46, 47). In addition, von Willebrand Factor (VWF) promotes the recruitment of leukocytes to the endothelium and extravasation of neutrophils in a platelet-dependent manner (48, 49). Similar protective effects of minocycline on CNS microvasculature have been reported in the retina and brain of rodents, including models of brain subarachnoid hemorrhage, ischemia reperfusion injury, and brain edema (50–53).

Here, we have reported sex differences in the progression of disease in the PDGFB-depleted retinas and revealed that the vascular engorgement is more likely to occur in male mice than in female mice. In addition, we have also highlighted inter- and intramouse differences in the retinal vascular phenotype. The progression of the vascular phenotype in one eye but not in the other mimics the clinical progression of DR. Based on this observation, we confirm that postnatal inhibition of PDGFB/PDGFBRβ signaling reproduces key features of the human disease in adult mice. However, we suggest further investigation on the sex differences in *Pdgfb* gene deletion efficiency and disease outcome to unravel the molecular basis for the present findings. Although angiographic images showed that vascular tortuosity and dilation was more prevalent in the *Pdgfb*^iECKO^ than in the APB5 model, EC-specific depletion of Pdgfb did not reproduce a much worse disease phenotype in 4-week-old mice compared with the pericyte depletion with APB5. Although the PDFGB-depletion model is a genetic model, the pathology triggered by Cre-inducible lines is more dependent on factors such as the dose and route of tamoxifen administration, age of mice, and time point of analyses (54).

In summary, the findings of the present study show that minocycline confers protective effects in the mouse retina following the attenuation of PDGFB/PDGFRβ signaling. We have shown that minocycline downregulates the inflammatory gene signature of microglia, microglia reactivity, and secretion of angiogenic factors during disease pathogenesis. The dual effect of minocycline on both the vasculature and immune cells partially protects the retina from advanced pathology.

## Methods

### Sex as a biological variable.

All studies were carried out on mixed sex populations. Male and female mice were distinguished in adult mice (4 weeks old) but not in neonatal mice (P1–P10). All data from adult mice were investigated for sex-specific effects, and differences were found in the retinas of 4-week-old *Pdgfb*-depleted mice.

### Animals.

Specific pathogen–free (SPF) C57BL/6J mice were reared and bred at the Eye Clinic mouse container at the University Hospital Cologne, University of Cologne. The B6.129P2-Pdgfb^tm2Cbet^/J strain commonly referred to as *Pdgfb*^fl/fl^ (stock no. 017622) (31) mice were purchased from The Jackson Laboratory. VE-Cadherin–Cre-ER^T2^ mice were generated as described previously (55). Mice were housed under SPF conditions in individually ventilated cages (GM 500, Tecniplast Greenline) with a maximum number of 5 adult mice in each cage. Room lighting was adjusted to a 12-hour/12-hour light/dark cycle with the light on at 6 a.m. and off at 6 p.m. The temperature and relative humidity were regulated to 22°C ± 2°C and 45%–65%, respectively. Mice were fed an irradiated phytoestrogen-free standard diet for rodents (Altromin 1314; 59% carbohydrates, 27% protein, 14% fat) or a diet containing PLX3397 hydrochloride (MedChemExpress) mixed with standard cow to final concentration of 1,200 ppm and had access to food and water ad libitum.

### Pharmacological inhibition of pericyte recruitment.

For the C57BL/6J mice, we aimed at blocking the recruitment of pericytes to growing ECs since the retinal vasculature develops during postnatal life in mice. To achieve this, 30 μg of a rat anti–mouse PDGFRβ mAb dissolved in PBS was injected s.c. once at P1. The control group received the same amount of a functional-grade rat IgG isotype dissolved in PBS. Both the PDGFRβ and rat IgG isotype control (catalogs 16-1402-82 and 16-4321-82, respectively), were sourced from Thermo Fisher Scientific. The proteins were concentrated to 30 μg using a 50k molecular weight cut-off (MWCO) protein concentrator (Thermo Fisher Scientific, 88540), and protein concentration was determined with BCA assay (Thermo Fisher Scientific, 23227).

### Tamoxifen injections.

Tamoxifen powder (T5648, Sigma-Aldrich) was partially dissolved in 100% ethanol and vortexed for 5 minutes. Heat-sterilized corn oil (C8267, Sigma-Aldrich) was added in a 9:1 (oil/ethanol) mixture ratio to a final concentration of 20 mg/mL TAM and incubated at 37°C until full dissolution. The corn oil was heat sterilized in an oven at 160°C and allowed to cool to room temperature. The prepared tamoxifen working solution was stored at –20°C protected from light and only diluted further in sterile corn oil to a concentration of 5 mg/mL. To induce Cre recombinase activity in the Cre-ER^T2^ mice, 100 μg of tamoxifen were injected s.c. daily from P5 to P7. Cre-ER^T2^ negative but fl/fl-positive mice (mice lacking expression of the Cre-ERT2 transgene but expressing the PDGFB floxed alleles) among the littermates were defined as WT mice for each experiment.

### PLX3397 diet.

Mice were fed with a diet containing PLX3397 (Plexxikon), mixed with normal diet chow at a concentration of 1,200 ppm for 7 days, starting at weaning (P21), and analysis was conducted at P28. Mice in the comparison groups were fed the regular diet.

### Visual acuity testing.

Analysis of visual acuity was performed with the OptoDrum device (StriaTech). Adult mice (4 weeks old) were placed on an elevated platform surrounded by computer monitors and a camera to observe animal behavior from above. The optomotor reflex was triggered with a black-and-white stripe pattern that rotated around the animal from the monitors. The stripe pattern is enhanced from wide stripes to much finer stripes, eventually reaching the threshold of the animal’s vision and the point at which the maximum reflex is achieved and maintained constantly. At this point, the OptoDrum software registers the visual acuity of the animal automatically.

### FA and spectral domain-optical coherence tomography.

Four-week-old mice were anesthetized with a mixture of ketamine (100 mg/kg bw, Ketavet; Pfizer Animal Health) and xylazine (2% Rompun; Bayer, 5 mg/kg bw) diluted in 0.9% sodium chloride by i.p. injection. Topical application of a drop of 2.5% phenylephrine and 0.5% topicamide was used to dilate pupils. Following anesthesia and pupil dilation, mice received i.p injections of 100 μL of 2.5% fluorescein (Alcon) diluted in 0.9% sodium chloride. Early-phase angiograms were recorded within 1 minute of fluorescein injection using Spectralis HRA/OCT (Heidelberg Engineering). SD-OCT was used to measure retinal thickness and the Heidelberg Eye Explorer (HEYEX) software was used to construct retinal thickness heatmaps within diameters of 3 and 6 mm from the optic nerve. The average of the 4 sectors surrounding the optic nerve within the 3 or 6 mm diameter accounted for 1 value of thickness.

### IHC of retinal flat mounts and sections.

Enucleated eyeballs from P10 and P28 mice were fixed in 4% PFA at room temperature for 1.15 hours and 2 hours, respectively. For whole-mount staining, retinas were dissected carefully and permeabilized before blocking unspecific binding sites with Perm/Block buffer overnight at 4°C. Next, whole mounts were incubated with selected primary antibodies diluted in Perm/Block buffer in the following dilutions: 1:500 for polyclonal rabbit anti-Iba-1 (019-19741, Wako) and monoclonal rat anti-CD31 (550274, BD Pharmigen); and 1:200 dilution of a polyclonal rabbit anti-NG2 antibodies (AB5320, Merck Millipore) overnight. After washing 3× with PBST-X (0.3% Triton X-100 in 1× PBS), tissues were further incubated with secondary antibodies (1:1,000 diluted in PBST-X) for 1 hour at room temperature protected from light. In addition, the retinal flat mounts were stained with TRITC-conjugated Isolectin B4 from *Bandeiraea simplicifolia* (1:100 diluted in Perm/Block, L5264, Sigma-Aldrich) for 1 hour at room temperature. After several washing steps in PBST-X, the retina and RPE flat mounts were prepared in DAKO mounting medium and allowed to dry at room temperature before microscopy.

To prepare cryosections of retinal tissue, fixed eyes were dehydrated by placing them in a graded sucrose concentration series of 10%, 15%, and 20% for the eyes from P10 pups, and 10%, 20%, and 30% for eyes from 4-week-old mice. Next, eyes were embedded in a cryomold filled with Tissue-Tek optimal cutting temperature (OCT, Sakura Finetek) medium and subsequently placed on dry ice. Retinal vertical sections of a thickness of 10 μm were cut using a cryostat (Leica, CM3050S) and used immediately for ISH RNAScope experiments or preserved at –20°C until staining. For processing of retinal sections, slides were allowed to thaw at room temperature for 10 minutes before hydration in 1× PBS. Furthermore, unspecific antigens were blocked with BLOTTO buffer for 30 minutes at room temperature followed by incubation with primary antibodies (diluted in antibody solution 1:500) at 4°C overnight. After washing steps, the sections were incubated with secondary antibodies (1:1,000 diluted in 1× PBS) at room temperature for 1 hour and protected from light. The antibodies used in the study are listed in Supplemental Table 2.

### RNAScope ISH.

RNAScope ISH (ACD, RNAscope Multiplex Fluorescent Reagent Kit v2) procedure was carried out with some modifications following the manufacturer’s instructions. Briefly, fresh frozen sections were pretreated with Protease Plus for 30 minutes at 40°C in a hybridization oven (HybEZ, ACD hybridization system) followed by washing thrice in cuvettes containing distilled water at room temperature. All the hybridization, amplification, and HRP blocking and signal detection steps were performed following the protocol provided by ACD. The following probes were used in this study: Mm-*Aif1*-C3, ACD 319141; Mm-*Vegf*-ver2-C1, ACD 412261; Mm-*Pgf*-C1, ACD 405921; and Mm-*Fgf2*-C1, ACD 316851. The C1 probes were labeled with TSA Plus Fluorophore Cyanine 5, while the C3 probes were labeled with TSA Plus Fluorophore Cyanine 3. Additionally, immunofluorescence staining was performed after ISH.

### RNA isolation, cDNA synthesis, and qPCR.

RNA was isolated from retinal tissue using the RNeasy Micro Kit (Qiagen) according to the manufacturer’s instructions. Retinal samples for RNA-Seq were dissected and immediately stored in RNAlater solution (Thermo Fisher Scientific) overnight at 4°C then frozen at –80°C and shipped on dry ice to the sequencing facility. In brief, after thawing and centrifuging for 5 minutes at 5,000*g*, the RNAlater was removed and the sample was disrupted and homogenized in 350 μL RLT buffer containing 1% β-mercaptoethanol with Precellys CK14 ceramic beads (1 cycle of 15 seconds at 5500 rpm) using a Precellys 24 Homogenisator (Bertin Corp.). Subsequently, the sample was centrifuged for 2 minutes at full speed, and 350 μL of the cleared supernatant was transferred to a new tube. One volume of 70% ethanol was added, and the sample was applied to an RNeasy MinElute spin column followed by an on-column DNase digestion and several wash steps. Finally, total RNA was eluted in 14 μL of nuclease-free water. The purity and integrity of the RNA were assessed on the Agilent 2100 Bioanalyzer with the RNA 6000 Nano LabChip reagent set (Agilent).

The first-strand complementary DNA (cDNA) was synthesized from the total mRNA using the RevertAid H Minus First-strand cDNA Synthesis Kit (Thermo Fisher Scientific) on a thermal cycler. Each reaction mix comprised 11 μL RNA, 0.2 μg/mL random hexamer primer,1× reaction buffer, 1 U/mL Ribolock RNAse inhibitor, 1 mM dNTP mix and 0.1 U/μL RevertAid RNA transcriptase. Synthesized cDNA was diluted with ddH2O to a working concentration of 20 ng/μL and 10 ng/μL for P10 and P28 retinas, respectively. The transcript levels of the genes of interest (Supplemental Table 3) were analyzed by qPCR performed in LightCycler 480 II (Roche) with SYBR Green (Takyon No Rox SYBR Master Mix dTTP blue, Eurogentec) technique. ATP synthase, H+-transporting, and mitochondrial F1 (*Atp5b*) were used as the housekeeping gene. Measurements were done in technical duplicates, and the expression of indicated genes was normalized to expression of *Atp5b* using the 2^ΔΔCT^ method.

### RNA-Seq.

Library preparation and RNA-Seq were carried out as described in the Illumina “Stranded mRNA Prep Ligation” Reference Guide, the Illumina NextSeq 2000 Sequencing System Guide (Illumina, Inc.), and the KAPA Library Quantification Kit - Illumina/ABI Prism (Roche Sequencing Solutions). In this procedure, 200 ng of total RNA was used for purifying poly-A–containing mRNA molecules using oligo(dT) magnetic beads. The mRNA was fragmented into small pieces, with an average size of 200–400 bases, using divalent cations at an elevated temperature of 94°C for 8 minutes. These RNA fragments were reverse transcribed into first-strand cDNA using reverse transcriptase and random hexamer primers. The inclusion of Actinomycin D ensured RNA-dependent synthesis, which improved strand specificity and prevented spurious DNA-dependent synthesis. Subsequently, the second-strand cDNA was synthesized using DNA Polymerase I, RNase H, and dUTP nucleotides. dUTP was incorporated instead of dTTP to prevent further amplification during PCR. The resulting cDNA fragments were adenylated at the 3′ ends, and the preindex anchors were ligated. Finally, DNA libraries were created using a 15 cycles of PCR to selectively amplify the anchor-ligated DNA fragments and to add the unique dual indexing (i7 and i5) adapters. The libraries were bead purified twice and quantified using the KAPA Library Quantification Kit. Equimolar amounts of each library were sequenced on an Illumina NextSeq 2000 instrument controlled by the NextSeq 2000 Control Software (NCS) v1.4.1.39716, using two P3 Flow Cells each run for 50 sequencing cycles with the dual index, single-read (SR) run parameters. The Real-Time Analysis Software (RTA) v3.9.25 was used for image analysis and base calling, and the resulting .cbcl files were converted into .fastq files with the bcl2fastq v2.20 software.

### FASTQ file processing alignment and differential expression analysis.

The Illumina BaseSpace Sequence Hub was utilized to process the .fastq files obtained from Illumina sequencing. The mouse mm39 reference genome (.fasta file) and corresponding reference annotation file (.gtf file) were acquired from NCBI and uploaded onto BaseSpace Hub to build a custom genome using the Reference Builder application (Illumina). The DRAGEN RNA Pipeline (Illumina) on BaseSpace Hub was used to align all the .fastq files against the custom genome and generate BAM files. The DRAGEN differential expression tool was used to analyze the BAM files and identify DEGs and gene counts, with significance at *P* ≤ 0.05 and a log_2_ fold change of ± 1. The identified DEGs were further processed on DAVID bioinformatics resource (56, 57). Volcano plots were generated on GraphPad Prism.

### Image analysis.

Morphometric analyses of the retinal microglia were performed on 2D images on MotiQ, an ImageJ (Fiji) plugin. Retinal vascular density was measured and expressed as the percent of CD31^+^ or IB4^+^ area divided by the total measured area using the AngioTool software version 0.6a (58). Pericyte coverage was calculated as NG2^+^ area divided by total CD31^+^ area of the capillary plexus. Vessel diameter was measured with the scale bar applied as a global measurement feature on ImageJ (NIH). The particle analyzer and pointer tools of ImageJ were used to count caspase-3^+^ cells and Iba1^+^ cells on retina whole mounts and vertical sections. Fluorescein intensity was measured using Image J.

### Statistics.

Alignment of RNA-Seq .fastq files was performed on Illumina BaseSpace Hub using a custom mouse genome and the DRAGEN RNA pipeline application. Further processing and differential expression analyses were performed using the DRAGEN differential analysis application on Illumina BaseSpace Hub. All data were analyzed using GraphPad Prism (version 9). One-way ANOVA followed by Šídák’s multiple comparisons test was used to calculate the difference between treatment groups. Data are presented as mean ± SD. *P* < 0.05 was considered statistically significant.

### Study approval.

All the animal husbandry and experimental procedures were carried out following the German law on animal protection and the ARVO statement for the use of animals in Ophthalmic and vision research. The governmental body responsible for animal welfare in the state of Nordrhein-Westfalen (Landesamt für Natur, Umwelt und Verbraucherschutz Nordrhein-Westfalen, Germany) reviewed and approved the experimental protocols in the present study. The animal experiment approval was issued under license no. 84-02.04.2020.A010.

### Data availability.

The gene expression data and have been deposited in the NCBI GEO repository under the accession no. GSE296651. Values for all data points are reported in the Supporting Data Values file.

## Author contributions

UMK’s roles included conceptualization, formal analysis, data curation, investigation, methodology, visualization, and writing of the original draft. CM’s roles included RNA-Seq analysis, methodology, reviewing, and editing. RHA contributed generation of the VE-Cadherin-Cre-ERT2 mice (46) on C57BL/6J background, proofreading, and editing. TL’s roles were conceptualization, funding acquisition, project administration, supervision, and editing the original draft as well as review.

## Supplementary Material

Supplemental data

Supporting data values

## Version 1 07/01/2025

In-Press Preview

## Version 2 08/08/2025

Electronic publication
