# Supplementary material for: Immunomodulation of inflammatory responses preserves retinal integrity in murine models of pericyte-depletion retinopathy
Source: JCI Insight. 2025 Jul 1;10(15):e184465. doi: 10.1172/jci.insight.184465 (PMC12333950; doi:10.1172/jci.insight.184465)
Supplement: Supplemental data [file jciinsight-10-184465-s226.pdf]

Supplemental material

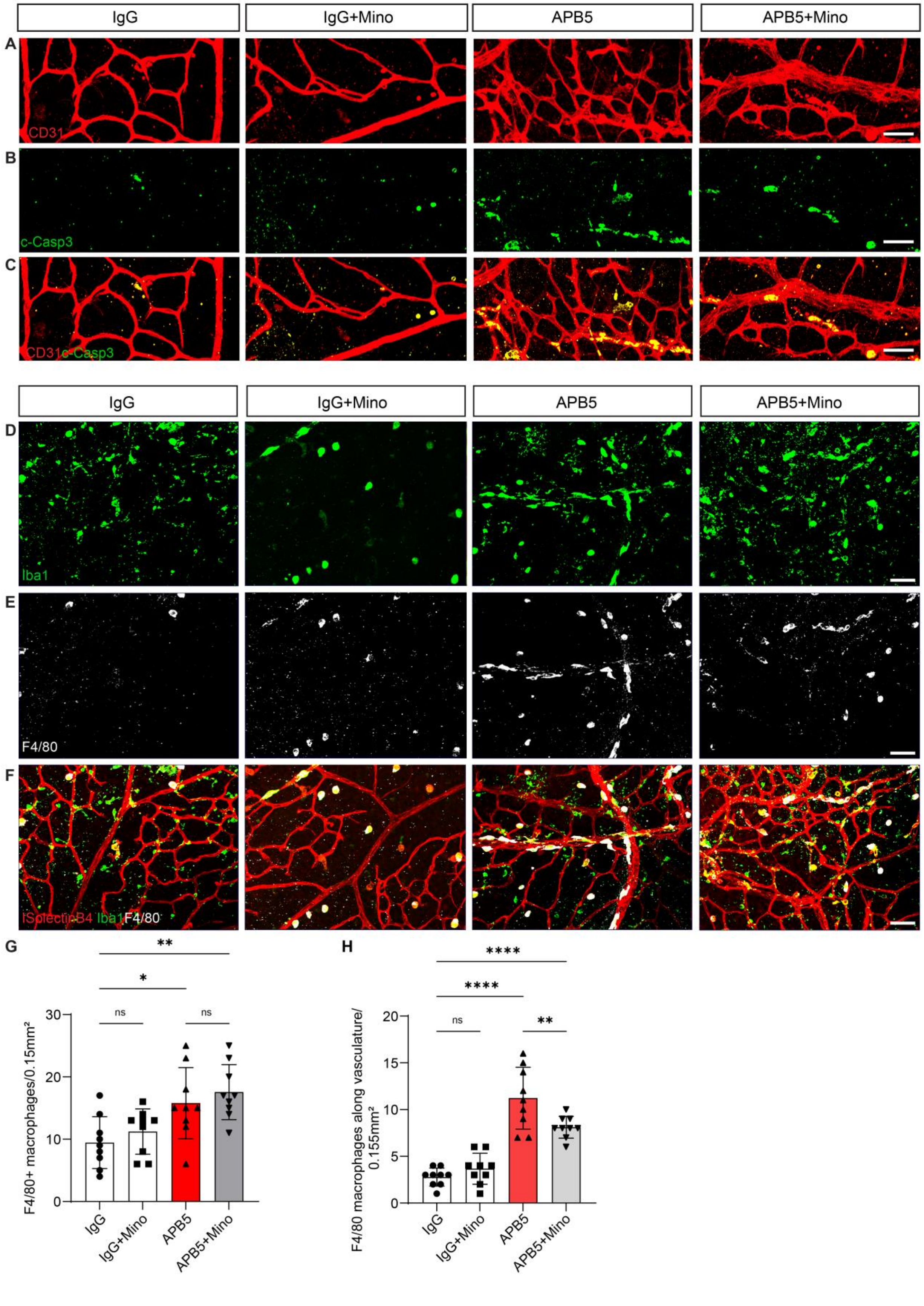

IgG
IgG+Mino
APB5
APB5+Mino
A
CD31
B
c-Casp3
C
CD31c-Casp3
D
Iba1
E
F4/80
F
IsolectinB4 Iba1 F4/80
G
F4/80+ macrophages/0.15mm²
ns
*
**
H
F4/80 macrophages along vasculature/ 0.155mm²
****
**

Supplemental Figure 1: A-C: High resolution images of cleaved caspase 3 and CD31 staining on regions of interest on retina flat mounts. D-F: Macrophage recruitment upon loss of iBRB integrity in P10 retinas. (D) Representative images of Iba1+ cells within the superficial plexus, coinciding with the ganglion cell layer. (E) Representative images of F4/80+ macrophages in the P10 retinas. (F) Representative merged channel images of Iba1, F4/80 and CD31 in p10 retinas. (G&H) Quantification of F4/80 macrophages per 0.15mm^2 of retina and along retinal vasculature (n = 9 from 3 -4 retinas each group). Scale bar is 50µm, *P < 0.05, **P < 0.01, ****P < 0.0001, ns; not significant. Data show mean ± SD. One-way ANOVA.

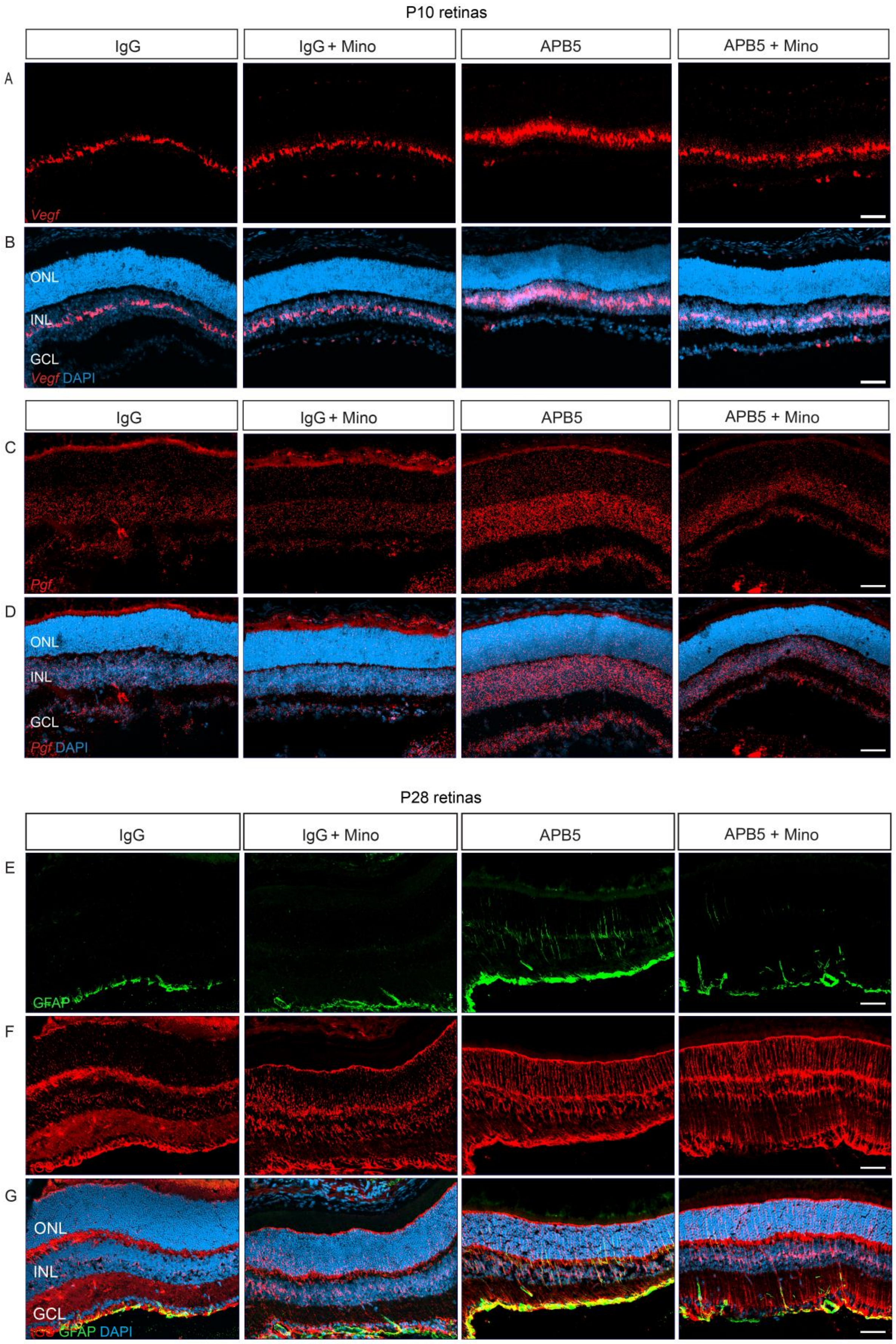

P10 retinas
IgG
IgG + Mino
APB5
APB5 + Mino
A
Vegf
B
ONL
INL
GCL
Vegf DAPI
IgG
IgG + Mino
APB5
APB5 + Mino
C
Pgf
D
ONL
INL
GCL
Pgf DAPI
P28 retinas
IgG
IgG + Mino
APB5
APB5 + Mino
E
GFAP
F
G
ONL
INL
GCL
GFAP DAPI

Supplemental Figure 2: A&B: Representative images of in situ hybridization of *Vegf* mRNA in retinal sections of IgG and APB5 treated mice with or without minocycline. **C&D:** Representative images of ISH of *Pgf* mRNA in retinal sections of IgG and APB5 treated mice with or without minocycline at P10. E-G: Representative images from IHC in 4 weeks-old retinas for GFAP (**E**) , GS (**F**) on retinal sections of 4-week old mice and (**G**) Merged channel images of both GFAP (green) and GS (red). GFAP: Glial fibrillary acidic protein, GS; Glutamine synthetase. Scale bar is 50μm.

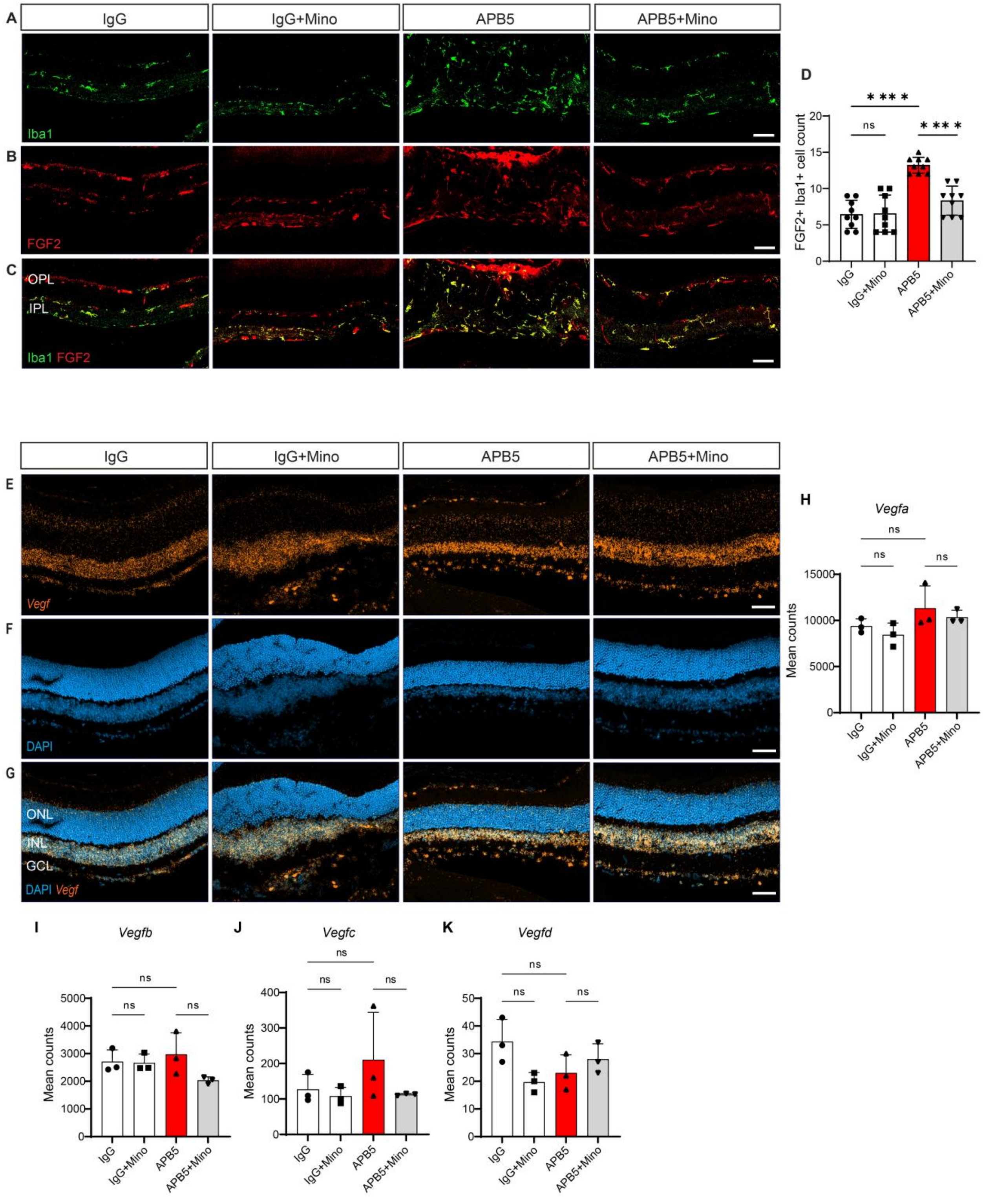

Supplemental Figure 3: APB5-mediated cellular effects in the mature retina. (**A-C**) Representative single channel images of Iba1 (**A**) and FGF2 (**B**) on retina sections. (**C**) Merged channel images of Iba1 (green) and FGF2 (red). (**D**) Quantitative analyses of Iba1$^+$FGF2$^+$

microglia in mature retinas. (**E**) Visualization of *Vegf* mRNA by ISH in IgG or APB5 retinas with or without minocycline treatment. (**F**) Representative single channel images of DAPI-stained nuclear layers. (**G**) Merged channels showing *Vegf* mRNA and DAPI stained retinal nuclear layers. (**H-K**) Quantification of the gene counts for *Vegf* isoforms in retinas of 4-week old mice. Scale bar is 50µm. ****$P < 0.0001$, ns; not significant. Data show mean ± SD. One-way ANOVA. FGF2; fibroblast growth factor 2, Iba1; Ionized calcium binding adapter molecule 1, Vegf; vascular endothelial factor.

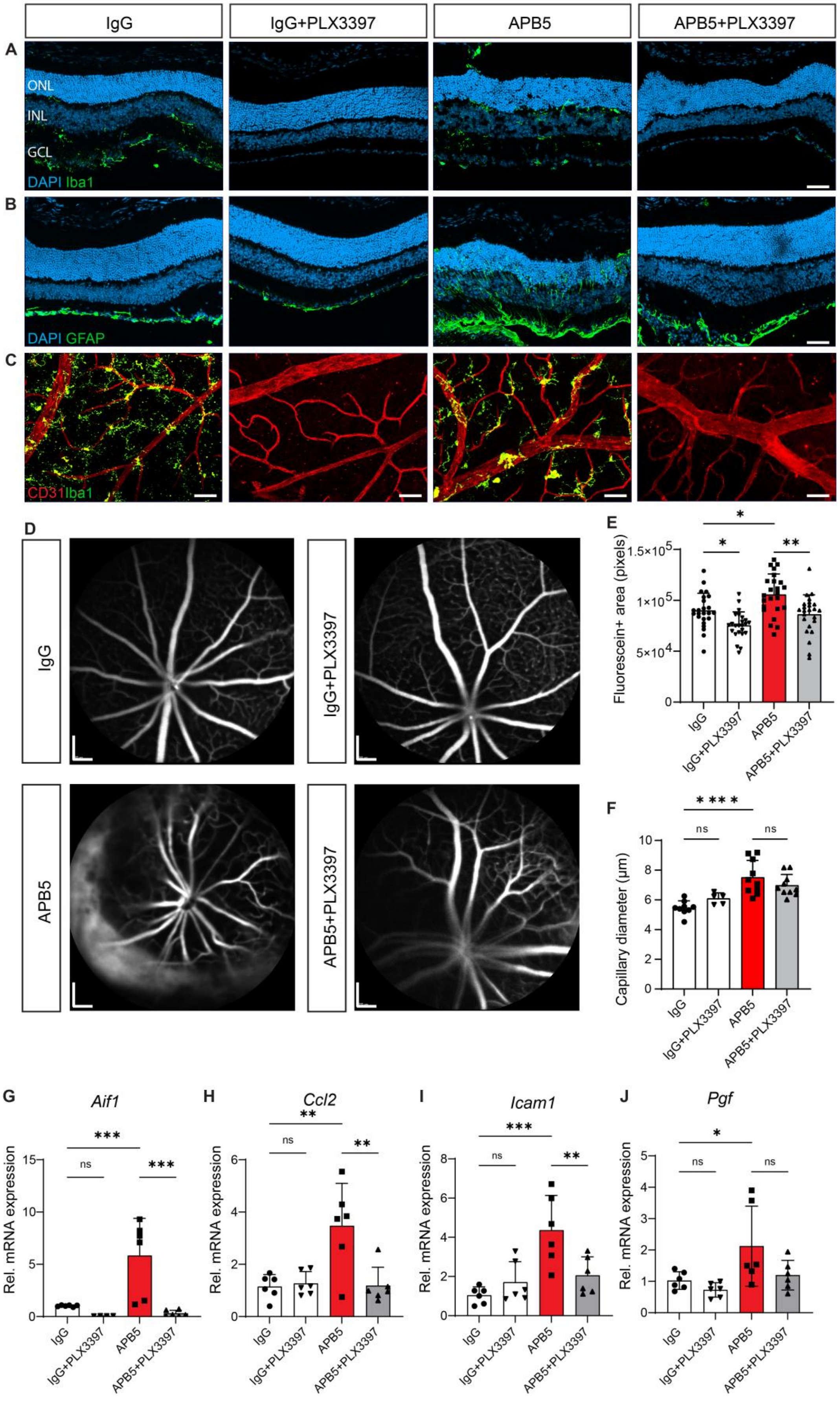

IgG
IgG+PLX3397
APB5
APB5+PLX3397
A
ONL
INL
GCL
DAPI Iba1
B
DAPI GFAP
C
CD31Iba1
D
E
Fluorescein+ area (pixels)
1.5×10^5
1×10^5
5×10^4
0
*
**
F
Capillary diameter (µm)
****
ns
G
Aif1
***
H
Ccl2
I
Icam1
J
Pgf
Rel. mRNA expression

Supplemental Figure 4: Depletion of CSF-1R expressing cells in retina with PLX3397. Mice in the PLX3397 groups were fed a diet containing PLX3397 (1,200 ppm) for 7 days starting at weaning (p21) and maintained for 7 days before analysis at 4-weeks of age (P28). (A&B) Representative images of retina cryo-sections stained for Iba1 and GFAP, respectively. (C) Representative merged images of CD31 and Iba1 staining of retina whole mounts in IgG and APB5-treated mice with or without administration of PLX3397. (D&E) Fluorescein angiographic images and indication of vascular permeability in 4-weeks old mice retinas (E; n = 24 eyes per group). (F) Measurement of capillary diameters across treated groups at 4 weeks of age (n = 5-10 flat mounts). (G-J) qRT-PCR analysis of mRNA levels of Aif1, Ccl2, Icam1 and Pgf in retinas of 4-weeks old mice (n = 6 retinas per group). A-C scale bar is 50µm, D, scale bar is 200 µm. *$P < 0.05$, **$P < 0.01$, ***$P < 0.001$,****$P < 0.0001$, ns; not significant. Data show mean ± SD. One-way ANOVA.

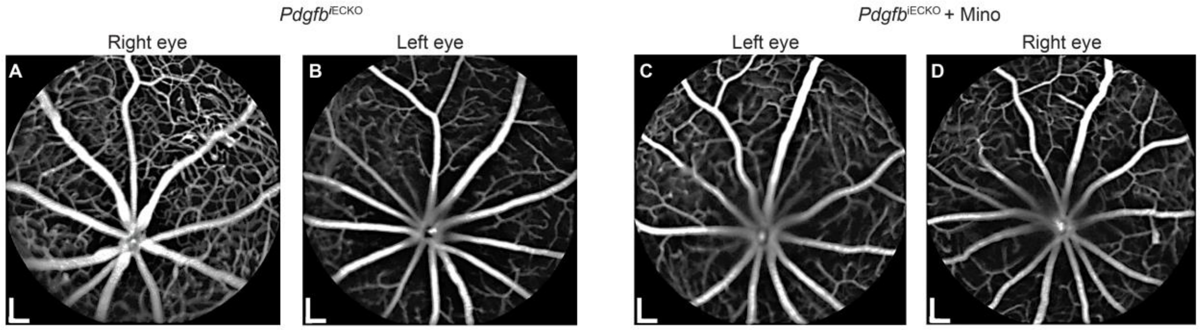

Supplemental Figure 5: Fluorescein angiographic images of *Pdgfb*-depleted retinas. (A&B) The right and left eyes from the same mouse showing pathology in the left eye only. (**C&D**) The right and left eye of the same mouse following treatment with minocycline. Scale bar is 200μm.

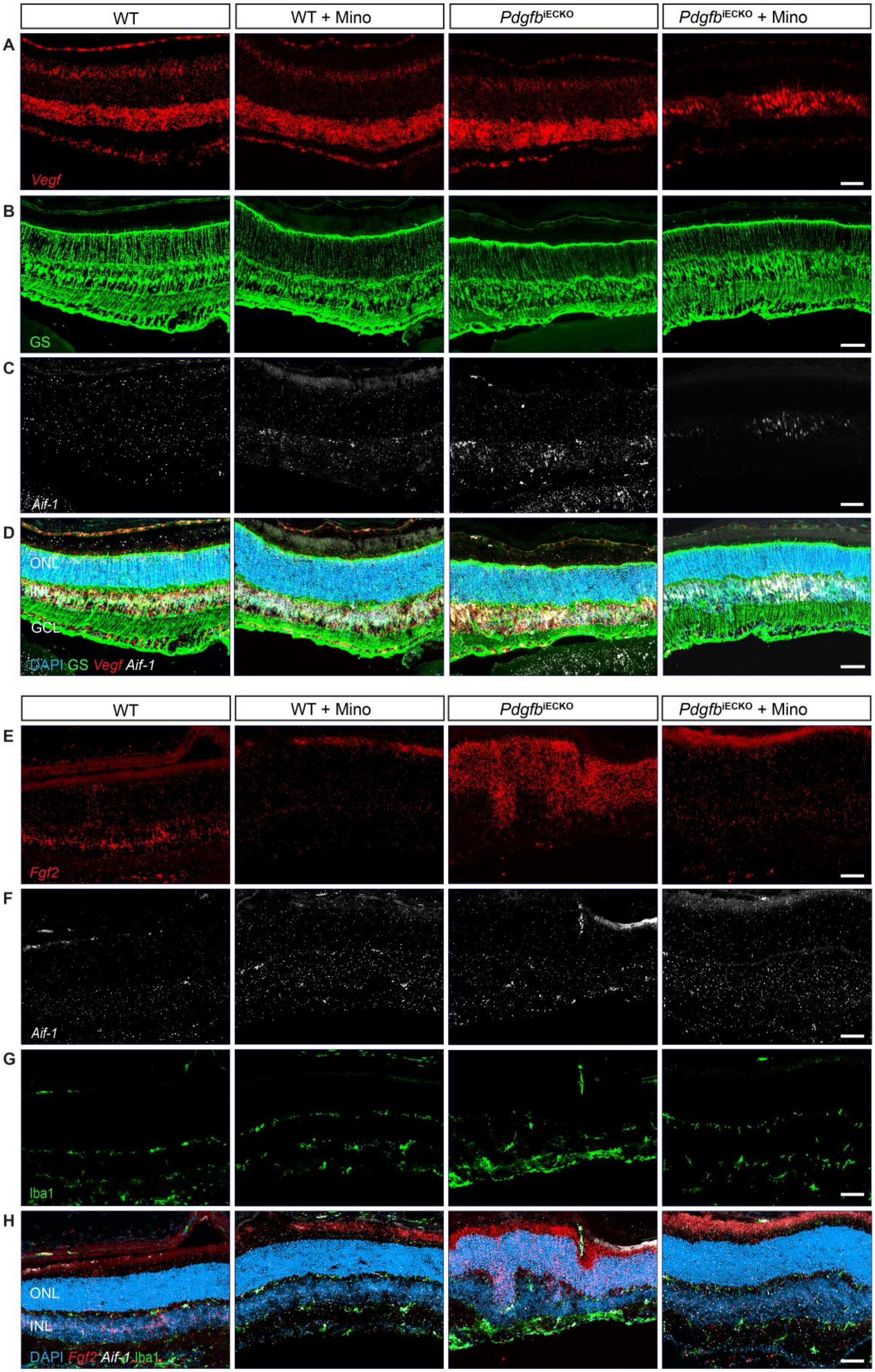
WT
WT + Mino
Pdgfb^iECKO
Pdgfb^iECKO + Mino
A
Vegf
B
GS
C
Aif-1
D
ONL
INL
GCL
DAPI GS Vegf Aif-1
WT
WT + Mino
Pdgfb^iECKO
Pdgfb^iECKO + Mino
E
Fgf2
F
Aif-1
G
Iba1
H
ONL
INL
DAPI Fgf2 Aif-1 Iba1

Supplemental Figure 6: Expression of *Vegf*, *Fgf2* and *Aif1* mRNA in *Pdgfb*-depleted retinas. **(A-C)** Representative images of ISH of *Vegf* mRNA (**A**), immunohistochemistry of GS (**B**) and ISH of *Aif1* mRNA(**C**) in retinal sections of 4-week old mice. (**D**) Merged images of Vegf, GS, Aif1 and DAPI. Scale bar is 50µm. GS (glutamine synthetase). (**E-G**) Representative images of ISH for Fgf2 (**E**), Aif1 (**F**) and immunohistochemistry for Iba1 (**G**). (**H**) Merged channel images showing the distribution of *Vegf*, *Aif1* mRNAs and Iba1 across retinal layers. Scale bar is 50µm.

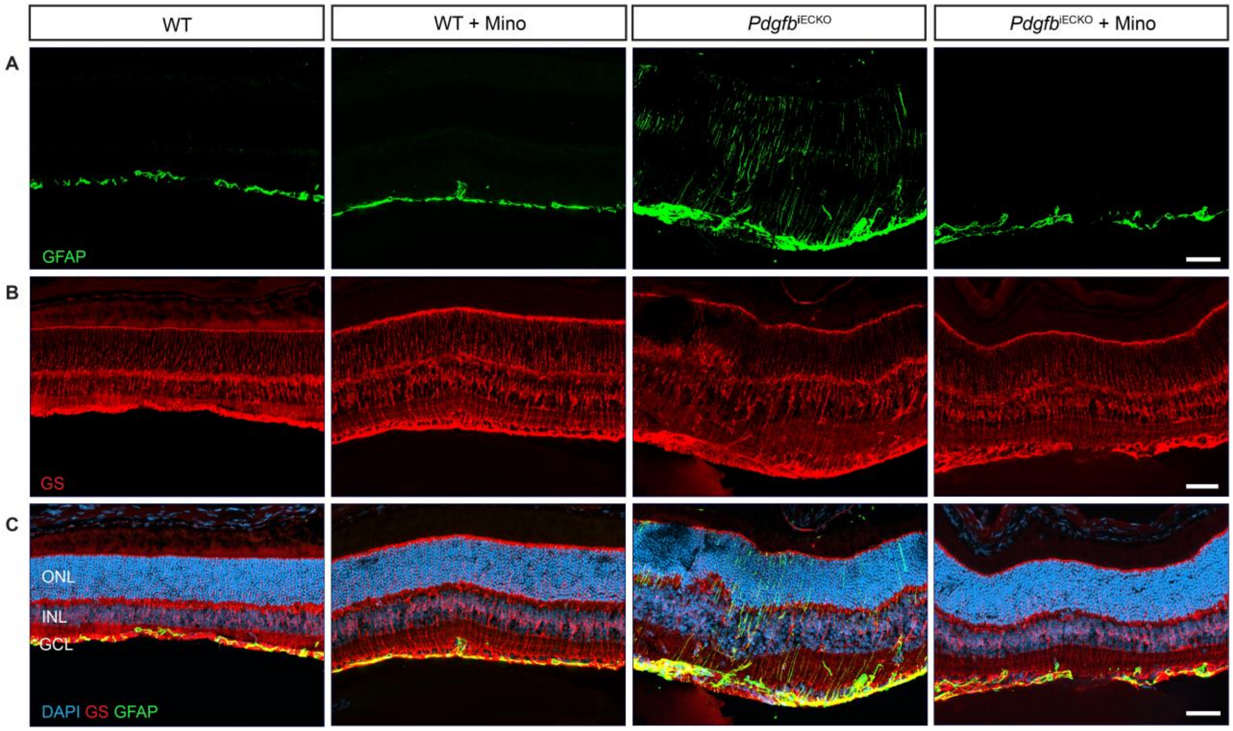

Supplemental Figure 7: Conditional loss of *Pdgfb* in retinal endothelium triggers astrocytic stress in the murine retina. (**A**) Representative IHC fluorescence images showing GFAP stress fibers in the retina. (**B**) Representative IHC fluorescence images of GS marker for Müller glia in the retina. (**C**) Merged channel images showing GFAP stress fibers and GS positive Müller glia. Scale bar is 50µm.

Supplemental Table S1: Scoring of vascular anomalies and fluorescein leakage in the *Pdgfb*^{iECKO} mice at four weeks of age using fluorescein angiography

	**Fluorescein leakage**		
**MALES**	**Right eye**	**Left eye**	**Both eyes**
M	yes	yes	yes
M	no	yes	no
M	no	yes	no
M	no	no	no
M	no	yes	no
M	yes	yes	yes
M	yes	yes	yes
M	yes	yes	yes
M	no	yes	no
M	yes	no	no
**% occurrence**	**50**	**80**	**40**
**FEMALES**			
F	yes	no	no
F	no	yes	no
F	no	yes	no
F	yes	optimal	no
F	no	yes	no
F	yes	yes	yes
F	yes	no	no
F	no	no	no
**% occurrence**	**50**	**50**	**12.5**

Supplemental Table S2: Antibodies and conjugated labels used in the study

Antibodies	Species/clonality	Dilution	Manufacture, Cat. No
Anti-CD31	Rat monoclonal	1:500	BD Pharmingen, 550274
Anti-NG2	Rabbit polyclonal	1:200	Merck Millipore, AB5320
Anti-Iba1	Rabbit polyclonal	1:500	FUJIFILM Wako, 019-19741
Anti-GFAP	Rabbit polyclonal	1:500	Sigma Aldrich, G9269
Anti-GS	Mouse monoclonal	1:500	Merck Millipore, MAB302
Anti-cleaved Caspase 3	Rabbit	1:250	Cell Signalling, 9661S
Anti-FGF2	Mouse monoclonal	1:200	Santa Cruz Biotechnology. Sc 74412
Anti-F4/80	Rat monoclonal	1:200	Origene, BM4007S, clone BM8
Alexa Flour 488	Donkey anti-rabbit polyclonal	1:1000	Thermo Fisher Scientific, A21206
Alexa flour 488	Goat anti-mouse	1:800	Thermo Fisher Scientific, A11001
Alexa Flour 594	Goat anti-rat polyclonal	1:1000	Thermo Fisher Scientific, A-11007
Alexa Flour 647	Donkey anti-rabbit	1:1000	Invitrogen, A-31573
TRITC-conjugated isolectin B4	*Bandeiraea simplicifolia*	1:100	Sigma-Aldrich, L5264,

Supplemental Table S3: List of SYBR® Green primers used in the study

Mouse Gene	Forward Primer (5' – 3')	Reverse Primer (5' – 3')	NM accession number
Acta2	gacaccacccacccagagt	acatagctggagcagcgtct	NM_007392.3
Actg2	ttcaggctgtgctctcactc	atggggacattgtgggtgac	NM_009610.2
Aif1	ggatttgcagggaggaaaag	tgggatcatcgaggaattg	NM_019467.3
Ang2	ccaccagtggcatctacaca	acccacgtccatgtcacag	NM_007426.4
Atp5b	ggcacaatgcaggaaagg	tcagcaggcacatagatagcc	NM_016774.3
Casp1	gctcaagttgacctcagagaaat	cacctctttcaccatctccag	NM_009807.2
Ccl2	catccacgtgttggctca	gatcatcttgctggtgaatgagt	NM_011333
Edn2	ctgccaaagcgttgtgagtg	cagcttcaggccagtgtctt	NM_007902.3

Fgf2	gcgacccacacgtcaaacta	ccgtccatcttccttcatagc	NM_008006.2
Glycam1	agctggtatgagccagggaag	ccactgtctggcttgacttg	NM_001289587.1
Icam1	cccacgctacctctgctc	gatggatacctgagcatcacc	NM_010493.3
Il-1β	agttgacggaccccaaaag	agctggatgctctcatcagg	NM_008361.4
Inos	ctttgccacggacgagac	tcattgtactctgagggctga	NM_001313922.1
Lgals3	gtgaaacccaacgcaaaca	ctcattgaagcgggggtta	NM_001145953.1
Lyz2	gaatggaatggctggctact	cgtgctgagctaaacacacc	NM_017372.3
Pgf	ctgggttggctgtgcatt	ggcaccacttccacttctgt	NM_001271705.1, NM_008827.3
Sema3g	gccagagccaaaacaaagcag	agtgtagtttcctgcgtcatgg	NM_001025379.1
Stat3	ctgtgtgacaccaacgacct	caatgaatctaaagtgcggggg	NM_213659.3
Tnf-α	ctgtagcccacgtcgtagc	ttgagatccatgccgttg	NM_013693.3
Tspo	actgtattcagccatggggta	accatagcgtcctctgtgaaa	NM_009775.4
Tyrobp	tctttctgcggccatgtcta	tgtgacgtccaaccaagtgaa	NM_011662.3
Vegfa	aaaaacgaaagcgcaagaaa	tttctccgctctgaacaagg	NM_001025250, NM_001317041
Vwf	cagagtctgagcagatccatcc	acctgaaagggttcatcttgcc	NM_011708.4
